# In utero exposure to threat of evictions and preterm birth: Evidence from the United States

**DOI:** 10.1111/1475-6773.13551

**Published:** 2020-09-25

**Authors:** Aayush Khadka, Günther Fink, Ashley Gromis, Margaret McConnell

**Affiliations:** ^1^ Department of Global Health and Population Harvard T. H. Chan School of Public Health Boston Massachusetts; ^2^ Swiss Tropical and Public Health Institute & University of Basel Basel Switzerland; ^3^ Department of Sociology Princeton University Princeton New Jersey

**Keywords:** adverse pregnancy outcomes, in utero exposure, preterm birth, threatened evictions

## Abstract

**Objective:**

To estimate county‐level associations between in utero exposure to threatened evictions and preterm birth in the United States.

**Data Sources:**

Complete birth records were obtained from the National Center for Health Statistics (2009‐2016). Threatened evictions were measured at the county level using eviction case filing data obtained from The Eviction Lab (2008‐2016). Additional economic and demographic data were obtained from the United States Census Bureau and Bureau of Labor Statistics.

**Study Design:**

We conducted a retrospective cohort analysis using 7.3 million births from 1,633 counties. We defined threatened eviction exposures as the *z*‐score of average case filings over the pregnancy and by trimester. Our primary outcome was an indicator for preterm birth (born < 37 completed weeks of gestation). Secondary outcomes included a continuous measure for gestational length, a continuous measure for birth weight, and an indicator for low birth weight (born < 2500 g). We estimated within‐county associations controlling for individual‐ and time‐varying county‐level characteristics, state‐of‐residence‐year‐and‐month‐of‐conception fixed effects, and a county‐specific time trend.

**Data Collection/Extraction:**

We merged birth records with threatened eviction data at the county‐month‐year level using mother's county of residence at delivery and month‐year of conception. We supplemented these data with information on county‐level annual 18‐and‐over population, annual poverty rate, and monthly unemployment rate.

**Principal Findings:**

Increased levels of eviction case filings over a pregnancy were associated with an increased risk of prematurity and low birth weight. These associations appeared to be sensitive to exposure in the second and third trimesters. Associations with secondary outcomes and within various population subgroups were, in general, imprecisely estimated.

**Conclusions:**

Higher exposure to eviction case filings within counties, particularly in the latter stages of a pregnancy, was associated with an increased risk of adverse birth outcomes. Future research should identify the causal effect of threatened evictions on maternal and child health outcomes.

1 **What is already known?**

- The threat of evictions has been increasing over the past two decades, particularly for low‐income Americans.
- Although a growing body of evidence shows that threatened evictions are associated with poor health outcomes, little is known about its relationship with maternal and child health outcomes.

2 **What does this study add?**

- We show that increased in utero exposure to eviction case filings was associated with an increased risk of prematurity and low birth weight over the study period.
- We find some evidence to suggest that risk of prematurity was sensitive to increased levels of threatened evictions in the second and third trimesters.

## 1 INTRODUCTION

The threat of evictions, defined as filing for a landlord‐initiated forced removal from a rental unit in local court, has become an increasingly common feature of the lives of low‐income Americans. In 2016, approximately 2.4 million eviction cases were filed in the United States.^1^ While this number represented approximately 6 percent of all renter‐occupied households in the same year, many cities, such as North Charleston, South Carolina, or Richmond, Virginia, experienced substantially higher risk of threatened evictions.^2^, ^3^

A developing literature links eviction to harmful effects on health. Several studies have documented associations between increased eviction prevalence and elevated risk of experiencing stress, depression, anxiety, psychological distress, and drug use.^4^, ^5^, ^6^, ^7^, ^8^, ^9^, ^10^, ^11^, ^12^ The evidence with respect to physical health outcomes is more mixed: Some studies have demonstrated positive associations between evictions and chronic disease prevalence and emergency room use^9^, ^13^; others, in contrast, have found no evidence of associations between evictions and poor health status.^4^, ^10^, ^14^

Despite mounting evidence of a link between health and eviction, there is less evidence on the relationship between threatened evictions and key maternal and child health outcomes. Evidence from the housing literature suggests that families with small children may be particularly likely to be threatened with evictions and, ultimately, be evicted.^4^, ^15^, ^16^, ^17^ Pregnant women and newborns threatened with eviction may be especially vulnerable to negative health effects as well.^18^ We aimed to fill this gap by estimating associations between in utero exposure to county‐level eviction filings and adverse birth outcomes, particularly preterm births. We also investigated whether these associations varied by pregnancy trimester. We analyzed preterm births as our primary outcome because it is the second largest contributor to infant mortality in the United States.^19^ Furthermore, preterm babies face significantly higher risk of long‐term morbidity and developmental challenges which lead to, among other things, substantially higher financial costs for all parties involved and a higher psychological toll for the caregivers.^20^, ^21^, ^22^, ^23^, ^24^

We hypothesized that increased prenatal exposure to threatened evictions would increase the likelihood of preterm birth and other adverse birth outcomes. This is because the threat of evictions is a key source of stress and other poor mental health outcomes, and a large literature demonstrates that high levels of prenatal stress are strongly associated with worse birth outcomes.^20^, ^25^, ^26^, ^27^, ^28^

## 2 METHODS

### 2.1 Study design and data

We conducted a retrospective cohort analysis by combining the largest county‐level dataset on legal eviction case filings in the United States to date with restricted‐use national birth records from the National Center for Health Statistics (NCHS).

Data on eviction filings were provided by The Eviction Lab at Princeton University and contained county‐month‐year‐level counts of the number of eviction cases filed in local court for 1633 counties between 2008 and 2016.^1^ Temporal coverage varied across counties. As a measure of the quality of the threatened eviction estimates, the data also identified counties for which the number of case filings in any county‐year fell within 85 percent‐115 percent of estimates obtained directly from the courts for the same county in the same year.

Live birth records from the NCHS represented the universe of live births in the United States between 2009 and 2016. These data contained individual‐level information on each woman's demographics, delivery payment method, self‐reported county of residence at delivery, completed weeks of gestation, birth weight, and day, month, and year of delivery.

We supplemented our analysis by using county‐level data from two other sources. From the United States Census Bureau, we used data on annual, county‐level 18‐and‐over population and annual, county‐level poverty rate.^29^ From the Bureau of Labor Statistics, we used information on monthly, unstandardized county unemployment rates.^30^

### 2.2 Exposure definition

For each pregnant woman, we constructed two exposure variables using data on county‐month‐year eviction case filings. The first exposure was defined over the duration of the pregnancy from the month of conception to the month of delivery. The second exposure was defined separately for the first trimester (month of conception to third month of gestation) and together for the second and third trimesters (fourth month of gestation to the month of delivery). We label the first exposure as EP (ie, exposure during pregnancy) and the second exposure as ET (ie, exposure by trimester).

To construct EP and ET, we estimated each woman's date of last menstrual period (LMP) using information on the obstetric/clinical estimate of gestational length and the day of week, month, and year of delivery. Next, we assigned a date of conception for each woman in our sample by assuming that conception occurs two weeks after the estimated date of LMP. Finally, we identified the number of eviction cases filed in each month of a woman's pregnancy by using information on her month‐year of conception and county of residence at delivery.

Having assigned eviction cases to each pregnant woman for each month of their pregnancy, we constructed EP and ET in three steps. First, we normalized the number of cases filed for each county‐month‐year by the county's 18‐and‐over population for the same year. Next, we estimated the average of the population‐normalized eviction case filings over the duration of each woman's pregnancy as well as separately for the first trimester and second and third trimesters combined. Finally, we standardized the average, normalized cases to define a z‐score (${\text{cases}}_{i,t}^{z\text{score}}$) as

(1) $${\text{cases}}_{i,t}^{z\text{score}}=\frac{{\text{cases}}_{i,t}^{\text{avg},\;\text{norm}}‐\bar{{\text{cases}}_{t}^{\text{avg},\;\text{norm}}}}{SD\left({\text{cases}}_{i,t}^{\text{avg},\text{norm}}\right)}$$

where ${\text{cases}}_{i,t}^{\text{avg},\text{norm}}$ represents the average normalized case filings for each pregnant woman *i* over duration of pregnancy/trimester *t*, $\bar{{\text{cases}}_{t}^{\text{avg},\text{norm}}}$ represents the mean of the average normalized case filings in the entire analytic sample over time period *t*, and SD(·) represents the standard deviation operator.

### 2.3 Outcome definition

We defined our primary outcome, preterm birth, as an indicator variable which equaled one if completed weeks of gestation for a newborn was reported as less than 37 weeks and zero otherwise. We used the obstetric/clinical estimate of gestation as our preferred measure for gestational length following NCHS recommendations.^31^

We also estimated associations between threatened evictions and three secondary pregnancy outcomes: (1) completed weeks of gestation defined as a continuous variable and measured using the obstetric/clinical estimate; (2) birth weight (in grams) defined as a continuous variable; and (3) low birth weight defined as an indicator variable which equaled one if birth weight was less than 2500 g and zero otherwise.

### 2.4 Analytic strategy

We constructed our analytic sample by restricting live birth observations based on the following inclusion criteria: (a) had obstetric/clinical estimate of gestation reported; (b) mother resided in a county for which eviction case filing data were available; (c) eviction case filing data were available for each month of gestation; (d) delivery occurred in a state that had adopted the 2003 revised birth certificate in the year of delivery; and (e) the live birth was singleton.

To prepare the data for analysis, we merged birth records with the eviction case filing data using information on the estimated month‐year of conception associated with each live birth and each pregnant woman's county of residence at delivery. We similarly merged supplementary datasets on county‐level population, unemployment, and poverty. Finally, for each observation in our data, we estimated eviction exposures EP and ET using Equation (1). We then constructed the analytic sample by applying the study inclusion criteria.

We computed descriptive statistics of the outcome and covariates of interest in the analytic sample by tertiles of exposure EP. We assumed that covariate data were missing at random and accounted for them by constructing five imputed datasets under the assumption that the observed and unobserved data together followed a multivariate normal distribution. In each imputed dataset, we winsorized exposures EP and ET at the 1st and 99th percentile to reduce the influence of extreme values of the exposure.^32^ We then estimated the following equation:

(2) $$y_{i,c,m,t}=\alpha +\beta z{\text{score}}_{i,c}+\lambda^{\prime }X_{i,c,t}+\delta_{c}+\theta_{s(c),t}+{\text{time}}_{c}+\epsilon_{i,c,m,t}$$

In Equation (2), β represents the association between eviction filings and adverse birth outcomes, $X_{i,c,t}$ represents individual‐ and county‐level covariates, $\delta_{c}$ represents county fixed effects defined based on each woman's self‐reported county of residence at delivery, $\theta_{s(c),t}$ represents state‐of‐residence‐year‐and‐month‐of‐conception fixed effects, and ${\text{time}}_{c}$ represents a county of residence–specific linear time trend. Equation (2) allows us to make within‐county contrasts while flexibly controlling for temporal trends in the outcome at the state and county levels. The individual‐level covariates we controlled for in this specification were mother's age, a quadratic age term, race, highest level of education, parity, child's sex, and method of payment for delivery. Although tobacco use during pregnancy or gestational diabetes is known risk factor of adverse birth outcomes, we did not control for these variables since they plausibly lie on the causal pathway between threatened eviction exposure and birth outcomes. At the county level, we controlled for a county's urban‐rural classification based on the NCHS classification system, average unemployment rate of each woman's county of residence over the duration of the pregnancy, and poverty rate of the county of residence for the year of conception.^33^, ^34^ The NCHS classifies counties into six urban‐rural categories: large central metro, large fringe metro, medium metro, small metro, micropolitan, and noncore.^33^, ^34^

We estimated Equation (2) using linear probability models (LPM) for the preterm birth and low birth weight outcomes. Although these are binary outcomes, we use the LPM because the model provides unbiased estimates of the marginal association between eviction filings and the outcome averaged over the distribution of the exposure variable.^35^ We also used ordinary least squares to estimate associations of the eviction case filing exposure with gestational length and birth weight. We accounted for correlated outcomes within counties by using Huber's cluster‐robust standard errors at the county level across all regression models.^36^ Finally, we pooled estimates of the coefficient and standard errors across the five imputed datasets using Rubin's rules.^37^

We assessed the robustness of our results in several ways. We defined a separate exposure variable using data on eviction filings in the nine months prior to conception and used this exposure as a negative control to conduct a falsification test.^38^ Evidence of a relationship between eviction filings and pre‐date pregnancy and pregnancy outcomes might indicate that our analytical model is identifying spurious relationships or pre‐existing trends between county‐level filings and our outcomes of interest. To assess whether our results are sensitive to exposure misclassification, we restricted the eviction case filing data to only those counties for which annual reported case filing counts were between 85 percent and 115 percent of external estimates (“verified cases”). Furthermore, to determine whether our association estimates are affected by the lack of complete county time series, we restricted the analytic sample to women who lived in counties for which we had a complete panel (“complete time series”). Finally, we restricted our analytic sample to counties that had eviction data for five or more years over the study period (“five‐year time series”). In all robustness tests, we imputed the missing data following the same procedure as in our primary analysis, winsorized the relevant exposure variables at the 1st and 99th percentiles, and estimated Equation (2).

To check for association heterogeneity, we separately re‐estimated Equation (2) in the analytic sample among white non‐Hispanic women, black non‐Hispanic women, Hispanic women, women of other races, and women who paid for their deliveries using Medicaid. We used payment for deliveries using Medicaid as a proxy for being low income. We winsorized exposures EP and ET at the 1st and 99th percentiles within each subgroup and estimated Equation (2).

### 2.5 Software

We used Stata/MP 15.1 to clean the data, conduct descriptive analyses, and estimate all regression models.^39^ We used “Amelia II” in RStudio to conduct multiple imputations of the analytic sample.^40^, ^41^

### 2.6 Ethical statement

This study was deemed to be exempt from human subjects review by the Office of Human Research Administration at the Harvard T. H. Chan School of Public Health.

## 3 RESULTS

**FIGURE 1:**
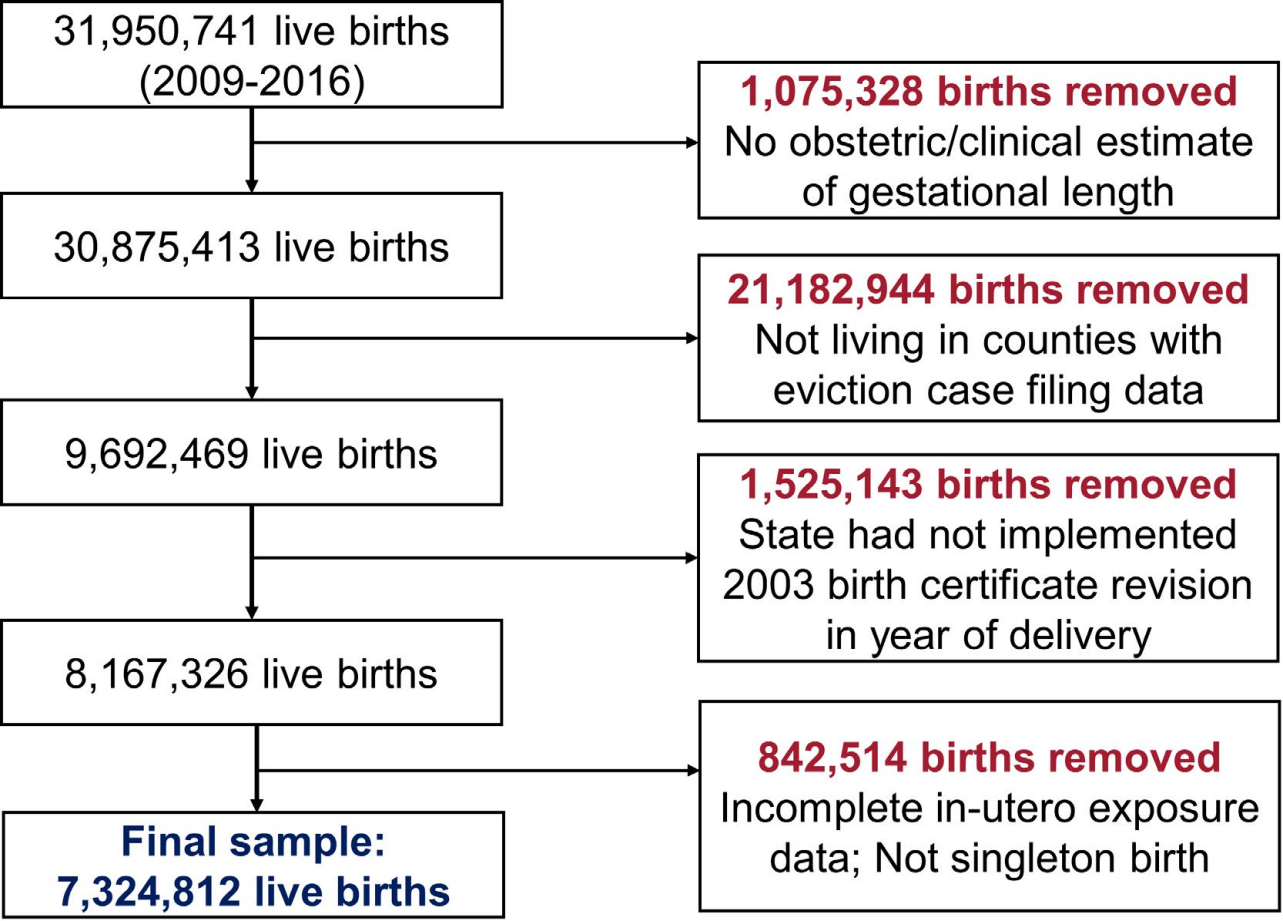
Construction of the analytic sample [Colour figure can be viewed at wileyonlinelibrary.com]

Our analytic sample consisted of 7 324 812 live births from 1633 counties in 39 states and the District of Columbia between 2009 and 2016. This sample was constructed from 31 950 741 live births across all counties in the United States over the same time period (Figure 1). The primary reason for not including live birth observations in the analytic sample was the lack of eviction case filing data at the county level. Figure S1 shows the states that were included in our analysis and the year from which they adopted the 2003 birth certificate revision, while Figure S2 shows the counties that appear in our analytic sample and the number of years for which we have eviction data for each county. Counties from the Midwest are best represented in our analytic sample in terms of their frequency and time series length. In contrast, we have relatively few counties from the northeast and the length of the time series of these counties is also relatively short. Finally, Table S1 shows that only six variables in our analytic sample had any item nonresponse and that the frequency of missing data in these six variables is very low.

**FIGURE 2:**
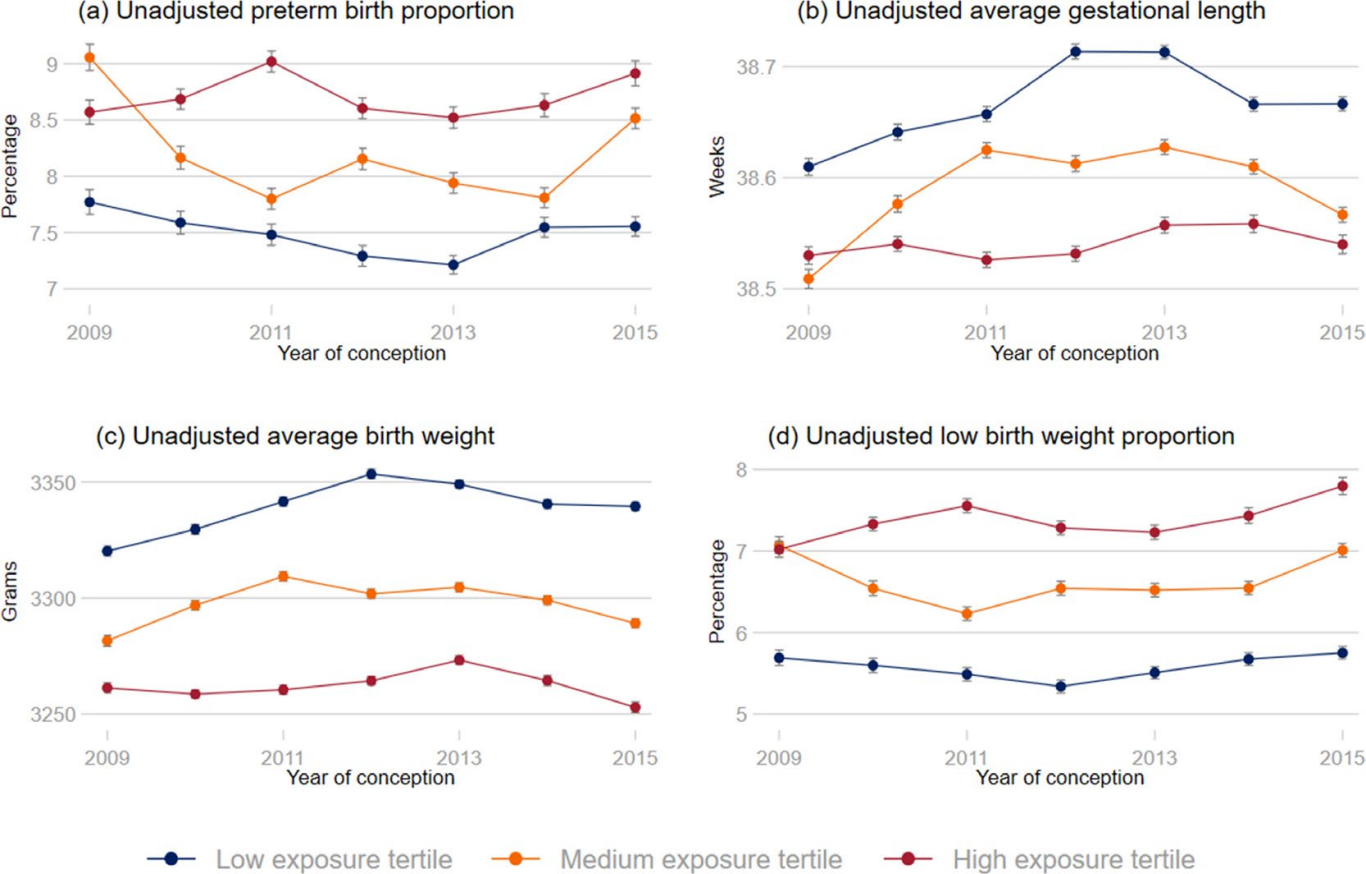
Unadjusted, annual average outcome by tertile defined using exposure to average eviction case filings over the duration of the pregnancy (exposure EP) and year of conception [Colour figure can be viewed at wileyonlinelibrary.com]

Figure 2 illustrates unadjusted, yearly averages of the four study outcomes by tertiles of exposure EP between 2009 and 2015. Panel (a) shows that the unadjusted preterm birth proportion was substantially lower among women who resided in counties with low levels of eviction filings (“low exposure tertile”) relative to women who resided in counties with high levels of eviction filings (“high exposure tertile”) in all years between 2009 and 2015. Similarly, panel (b) and panel (c), respectively, show that average gestational length and average birth weight were consistently higher in the low exposure tertile relative to the high exposure tertile over the same time period. Finally, panel (d) suggests that the proportion of low birth weight newborns was consistently lower in the low exposure group relative to the high exposure group between 2009 and 2015.

Differences in unadjusted average outcomes across exposure categories presented in Figure 2 may reflect underlying compositional differences in these groups. Table 1 presents differences in individual‐level socioeconomic characteristics and county‐level characteristics across the three tertiles of exposure EP. Relative to women in the low exposure group, a higher proportion of women in the high exposure group reported not having a high school degree. Similarly, the proportion of black non‐Hispanic and Hispanic women was substantially higher in the high exposure group relative to the low exposure group as was the proportion of women who reported paying for their deliveries using Medicaid. Counties represented in the high exposure group were also more likely to have higher unemployment and poverty rates, and to be classified as metropolitan relative to counties in the low exposure group. Table S1 shows that these differences between the high and low exposure groups were consistent over time.

**TABLE 1:** Distribution of individual‐ and county‐level covariates over the study period by tertiles defined using exposure to average eviction case filings over the duration of the pregnancy (exposure EP)

	Low exposure tertile	Medium exposure tertile	High exposure tertile
Individual level			
Mean age (y)	27.96	27.93	27.76
	(27.95‐27.96)	(27.92‐27.94)	(27.75‐27.77)
% of women with no high school	14.24%	16.59%	19.46%
	(14.2%‐14.29%)	(16.54%‐16.63%)	(19.41%‐19.51%)
% of women with high school but no tertiary degree	46.13%	46.74%	46.51%
	(46.06%‐46.19%)	(46.68%‐46.81%)	(46.45%‐46.57%)
% of women with a tertiary degree	39.63%	36.67%	34.03%
	(39.57%‐39.69%)	(36.61%‐36.73%)	(33.97%‐34.09%)
% White (non‐Hispanic)	72.02%	55.67%	40.61%
	(71.96%‐72.08%)	(55.61%‐55.73%)	(40.55%‐40.67%)
% Black (non‐Hispanic)	6.16%	17.36%	28.71%
	(6.13%‐6.19%)	(17.31%‐17.41%)	(28.65%‐28.76%)
% Hispanic	14.19%	21.08%	24.49%
	(14.15%‐14.24%)	(21.03%‐21.13%)	(24.44%‐24.55%)
% Other races	7.63%	5.89%	6.19%
	(7.6%‐7.66%)	(5.86%‐5.92%)	(6.16%‐6.22%)
% paying for delivery using Medicaid	38.29%	43.16%	44.67%
	(38.23%‐38.35%)	(43.1%‐43.23%)	(44.6%‐44.73%)
County level			
Average unemployment rate	6.70%	7.08%	7.61%
	(6.7%‐6.7%)	(7.08%‐7.08%)	(7.6%‐7.61%)
Average poverty rate	13.74%	15.73%	17.21%
	(13.73%‐13.74%)	(15.73%‐15.74%)	(17.2%‐17.21%)
% Metropolitan counties	65.95%	93.48%	97.58%
	(65.89%‐66.01%)	(93.45%‐93.51%)	(97.56%‐97.6%)

Note

95% confidence intervals in parentheses.

Table 2 presents our main results of the association between exposure EP and birth outcomes. We estimated that a one standard deviation increase in eviction case filings was associated with a 1.09 percentage point increase in the risk of preterm birth over the study period (β=1.09 percentage points; 95% CI: [0.05, 2.13]). Since the effective exposure variation is constrained by our primary specification—that is, we analyze the variation that remains after accounting for county‐level fixed effects, state‐of‐residence‐year‐and‐month‐of‐conception fixed effects, and a linear county‐level time trend—an association of 1.09 percentage points corresponds to a 0.08 percentage point increase, on average, in the risk of preterm birth (Figure S3). Relative to the sample average risk of preterm birth of 8.18 percent, a 0.08 percentage point increase in risk corresponds to an approximately 1 percent increase.

**TABLE 2:** Associations between exposure to average eviction case filings over the duration of the pregnancy (exposure EP) and birth outcomes

	(1)	(2)	(3)	(4)
Outcome	Preterm birth	Gestational length	Birth weight	Low birth weight
*Z*‐score of average case filing over pregnancy	1.09% points	−0.05 wk	−11.96 g	0.72% points
	(0.05‐2.13)	(−0.13‐0.03)	(−28.75‐4.82)	(0.02‐1.43)
Average value of the outcome in the analytic sample	8.18%	38.59 wk	3298.56 g	6.57%
Average value of the outcome in the United States over the study period	9.81%	38.49 wk	3269.06 g	8.07%
Observations	7 324 812	7 324 812	7 324 812	7 324 812

Note

95% confidence intervals in parentheses constructed using clustered standard errors at the county level. All results presented in the table are pooled estimates from ordinary least‐squares regressions estimated on five imputed datasets. All models controlled for county of residence fixed effects, state‐of‐residence‐year‐and‐month fixed effects, and a linear county‐specific time trend. All models also controlled for mother's age, a quadratic age term, mother's race, mother's highest level of education, parity, child's sex, method of payment for delivery, urban‐rural classification of county of residence, county of residence's annual poverty rate, and county of residence's unstandardized, monthly unemployment rate.

We also estimated a positive association between exposure EP and risk of low birth weight: A one standard deviation increase in average case filings over a pregnancy was associated with a 0.72 percentage point increase in risk of low birth weight (β=0.72 percentage points; 95% CI: [0.02, 1.43]). Given the effective variation we are working with, a 0.7 percentage point increase in risk corresponds to a 0.05 percentage point increase in the risk of newborns being born low birth weight (Figure S3). Associations between exposure EP and length of gestation as well as birth weight were negative but imprecisely estimated.

Table 3 presents associations between exposure ET and all study outcomes. In terms of the primary outcome, we estimated that a one standard deviation increase in threatened evictions in the second and third trimesters was associated with a 1.02 percentage point increase in the risk of preterm birth over the study period (β=1.02 percentage points; 95% CI: [0.015, 1.90]). After adjusting for the effective variation, this risk difference represented a 0.08 percentage point increase in the risk of preterm birth (Figure S4). Our estimate of the association between threatened evictions in the first trimester and preterm birth risk was imprecisely estimated.

**TABLE 3:** Associations between exposure to average eviction case filings by pregnancy trimester (exposure ET) and birth outcomes

	(1)	(2)	(3)	(4)
Outcome	Preterm birth	Gestational length	Birth weight	Low birth weight
*Z*‐score of average case filing in the first trimester	−0.06% points	0.004 wk	1.51 g	−0.01% points
	(−0.49‐0.37)	(−0.03‐0.04)	(−5.2‐8.21)	(−0.33‐0.31)
*Z*‐score of average case filing in the second and third trimesters	1.02% points	−0.05 wk	−11.70 g	0.69% points
	(0.15‐1.9)	(−0.13‐0.03)	(−26.76‐3.36)	(0.03‐1.34)
Average value of the outcome in the analytic sample	8.18%	38.59 wk	3298.56 g	6.57%
Average value of the outcome in the United States over the study period	9.81%	38.49 wk	3269.06 g	8.07%
Observations	7 324 812	7 324 812	7 324 812	7 324 812

Note

95% confidence intervals in parentheses constructed using clustered standard errors at the county level. All results presented in the table are pooled estimates from ordinary least‐squares regressions estimated on five imputed datasets. All models controlled for county of residence fixed effects, state‐of‐residence‐year‐and‐month fixed effects, and a linear county‐specific time trend. All models also controlled for mother's age, a quadratic age term, mother's race, mother's highest level of education, parity, child's sex, method of payment for delivery, urban‐rural classification of county of residence, county of residence's annual poverty rate, and county of residence's unstandardized, monthly unemployment rate.

In terms of the secondary outcomes, we estimated a positive association between threatened evictions in the second and third trimesters and risk of low birth weight (β=0.69 percentage points; 95% CI: [0.03, 1.34]). The estimated association corresponds to a 0.06 percentage point difference in the risk of low birth after accounting for the effective variation (Figure S4). The association between eviction case filings in the first trimester and the risk of low birth weight was imprecisely estimated. Associations with gestational length and birth weight for threatened evictions in the first trimester as well as the second and third trimesters were also imprecisely estimated.

Table S3 presents results from the falsification test using preconception exposure as a negative control and shows that associations of this variable with all four outcomes were small in magnitude and imprecisely estimated. Tables S4‐S6 present results from estimating Equation (2) using exposure EP in the three robustness samples and show that results from the verified cases subsample (Table S4) and the five‐year time series subsample (Table S6) were consistent with our main results.^1^ Tables S7‐S9 present results from estimating Equation (2) in the three robustness subsamples using exposure ET and show that results from the five‐year time series subsample (Table S9) are consistent with our primary results.

Results from analyses checking for association heterogeneity by racial subgroups and Medicaid payment status were largely consistent with the primary results (Tables S10‐S19). We estimated a positive association between prematurity and exposure EP among white non‐Hispanic women (Table S10). We also estimated a positive association between risk of being born low birth weight and exposure EP among white non‐Hispanic and black non‐Hispanic women (Table S13). Increased exposure to threatened evictions in the second and third trimesters was also associated with an increased risk of prematurity among women of other races (Table S15) and women who paid for their deliveries using Medicaid (Table S19). We also estimated a positive association between second and third trimester threatened eviction exposure and risk of delivering a low birth weight newborn among white non‐Hispanic women (Table S18).

## 4 DISCUSSION

In this study, we investigated the relationship between prenatal exposure to threatened evictions and adverse birth outcomes across 1633 counties in 39 states and the District of Columbia between 2008 and 2016. We also studied if these associations varied by pregnancy trimester. We found that increased exposure to eviction filings during a pregnancy was associated with an increased risk of prematurity and being born low birth weight. We also found some evidence to suggest that risk of preterm birth and low birth weight were particularly sensitive to eviction filings in the second and third trimesters of a pregnancy.

Results from various robustness checks largely support our primary results. Coefficients on the prepregnancy exposure variable, which we used as a negative control to conduct a falsification analysis, were small and imprecise. Although prepregnancy threatened eviction exposure may not be the ideal negative control because of its potential direct effect on birth outcomes through prepregnancy health, the falsification check results do provide suggestive evidence to support the claim that our primary results are not driven by spurious correlations. Results from estimating Equation (2) across the three robustness subsamples also support our primary results, especially for exposure EP. Finally, we were largely unable to estimate precise associations between threatened evictions and adverse birth outcomes by racial subgroups or among women who paid for their delivery using Medicaid insurance.

To the best of our knowledge, our study is one of the first to demonstrate that increased within‐county exposure to eviction filings increases the risk of preterm birth. The positive associations between eviction filings—a source of prenatal stress—and risk of prematurity and low birth weight are consistent with studies that have investigated the impact of prenatal stressors on pregnancy outcomes. For instance, Gemmill et al^27^ found that the election of Donald Trump as the president of the United States was associated with an increased risk of premature births among Latina women who were pregnant at the time of the election. Similarly, Currie et al^42^ demonstrated that exposure to assaults in utero was associated with a higher risk of adverse birth outcomes in New York City.

However, the associations we estimate for preterm birth risk and low birth weight risk have relatively wide confidence intervals. For the association between exposure EP and preterm birth, for example, our results are compatible with a 0.05‐2.13 percentage point increase in the risk of prematurity. This relatively wide range of estimates may in part reflect the ecological nature of our analysis which analyzes an exposure at the county level, that is, our analysis treats all pregnant women as exposed to a given level of eviction filings regardless of whether they personally received an eviction notice or were affected by one indirectly. This means that our associations likely capture some combination of the exposure of living in a community where evictions are common combined with the direct experience of receiving an eviction filing. Individual‐level data on eviction filings would allow for the estimation of these associations with a higher degree of precision and could consequently find substantially larger effects for pregnant women who were directly affected by the threat of evictions over the study period. The potential for community‐level effects of housing instability resulting from increased eviction filings would also be consistent with the housing and social epidemiology literatures which have shown the impact of neighborhood quality on health outcomes.^43^, ^44^, ^45^, ^46^

Another strength of our study is that we analyze the relationship between threatened evictions and physical health outcomes using data that have wide geographic and temporal coverage. Prior studies looking at associations between threatened evictions, actual evictions, and health outcomes have either come from other countries such as Spain or from very localized geographies within the United States.^4^, ^9^, ^10^, ^13^ Niccolai et al^47^ use similar data to estimate associations between evictions and sexually transmitted diseases across the country but limit their analysis to only 2014.

A final strength of our analysis is that it adds to the literature on the importance of the timing of in utero stressors on the risk of preterm birth and other pregnancy outcomes. In this regard, our finding regarding the relative importance of eviction case filing exposure in the second and third trimesters is consistent with some recent studies such as that by Gemmill et al.^27^ Earlier studies have suggested that exposure to traumatic events such as natural disasters in the first trimester is associated with poor birth outcomes although few previous studies have used national data and those that have were not focused on the United States.^48^, ^49^, ^50^, ^51^

A key limitation of our analysis is that we did not have complete geographic and temporal coverage in terms of the eviction case filing data. The United States has over 3000 counties, but we only had data from 1633. Lack of data from all counties is explained by the fact that collection of data on eviction cases is more difficult in some areas due to incomplete or nonstandardized electronic case management systems, limitations on access to paper case records, and restrictions on bulk records requests. It is unclear how these barriers to record collection are associated with case filing volumes, which limits the generalizability of our results. Furthermore, generalizability of our results may also be limited because we could only use approximately 57 percent of the possible 176 364 county‐month‐year observations, not only due to a lack of county‐level data but also because we restricted our analysis to those state‐years in which the 2003 birth certificate revision had been implemented.

Another limitation of our analysis is that we only observe residence at time of delivery, and we are therefore unable to determine whether a pregnant woman moved across counties over the course of her pregnancy. Movement across counties would mean that our exposure variable would be subject to measurement error that we expect would attenuate our results. However, there are reasons to believe that movement across counties may be limited in scope: The literature on evictions suggests that individuals who are evicted generally tend to move into worse quality neighborhoods and not necessarily across counties.^52^ A review of the residential mobility literature by Bell and Belanger^53^ also finds that there is some evidence on residential change during pregnancy which suggests that the distance moved is often very short (median distance < 10 km). In addition, a recent study by Garboden and Rosen^54^ also finds that threatened evictions do not always lead to actual evictions and therefore a change in residence.

A final limitation of our analysis is the possibility of selection bias due to exposure to higher levels of threatened evictions resulting in greater loss of fetuses that would have been born premature relative to lower levels of threatened eviction exposure.^55^, ^56^ We expect such fetal selection to attenuate our results.

## 5 CONCLUSIONS

The threat of evictions has been increasing over the past two decades, particularly for low‐income Americans. Our analysis shows that for pregnant women, higher levels of threatened evictions are associated with increased risk of adverse birth outcomes in general and premature deliveries in particular. Across the United States, several policies are currently being enacted to offset the threat of evictions—our study suggests that evaluating the causal impact of these policies on parental well‐being and child health is an important area of inquiry.

## Supporting information

Supplementary Material

Figure S1

Figure S2

Figure S3

Figure S4

Figure S5

Appendix S1

## ENDNOTE

^1^ The verified cases subsample included 7 027 351 live birth observations from 1632 counties across 39 states and the District of Columbia. The complete time series subsample consisted of 3 254 301 live birth observations from 617 counties from 30 states. Finally, the five‐year time series subsample had 6 364 818 observations from 1156 counties across 37 states.
