# Supplementary figures and images for: In utero exposure to threat of evictions and preterm birth: Evidence from the United States
Source: Health Serv Res. 2020 Sep 25;55(Suppl 2):823–32. doi: 10.1111/1475-6773.13551 (PMC7518827; doi:10.1111/1475-6773.13551)

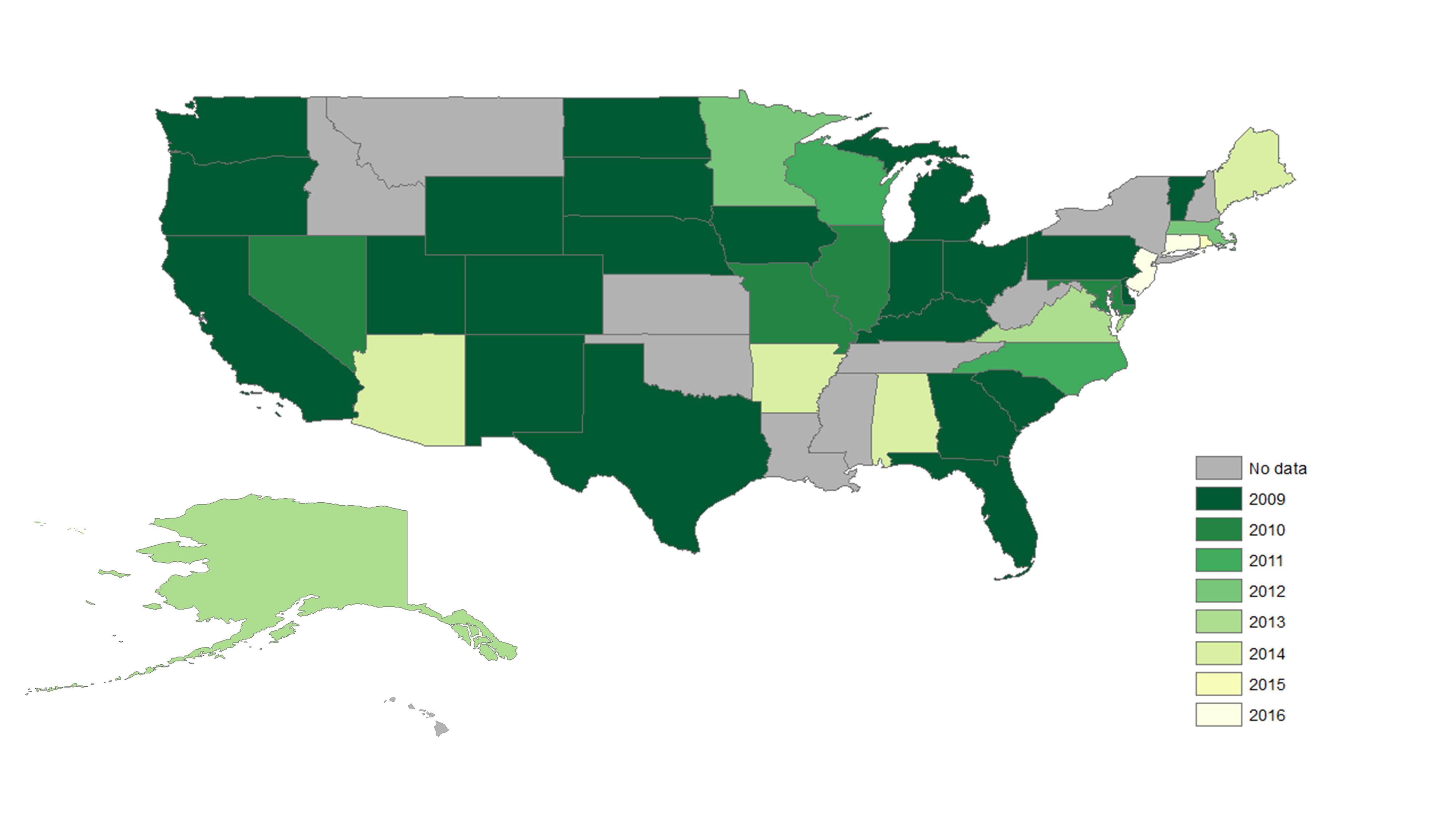

Supplement: Supplementary file 2 — Figure S1 [file HESR-55-823-s002.png]

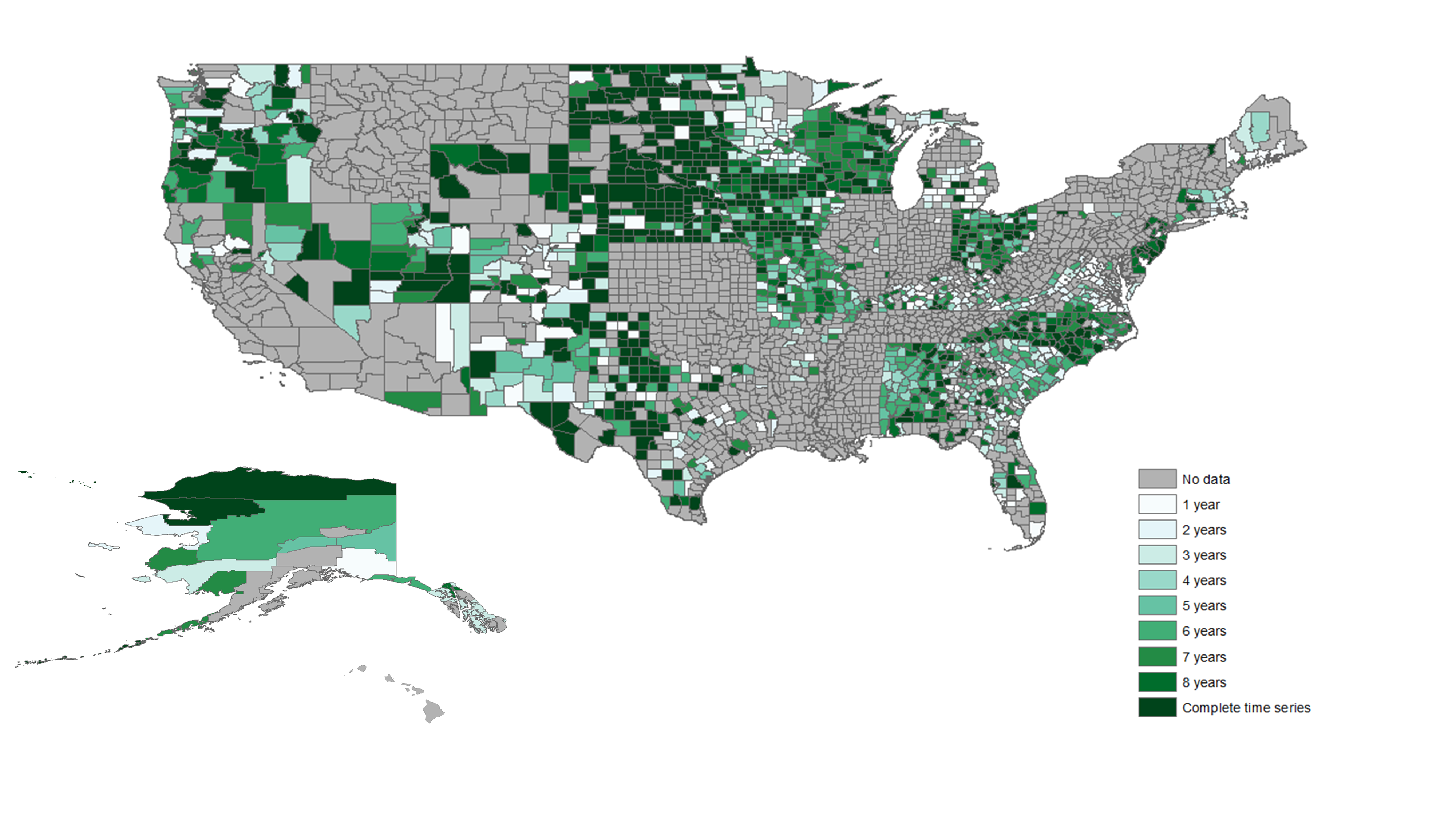

Supplement: Supplementary file 3 — Figure S2 [file HESR-55-823-s003.png]

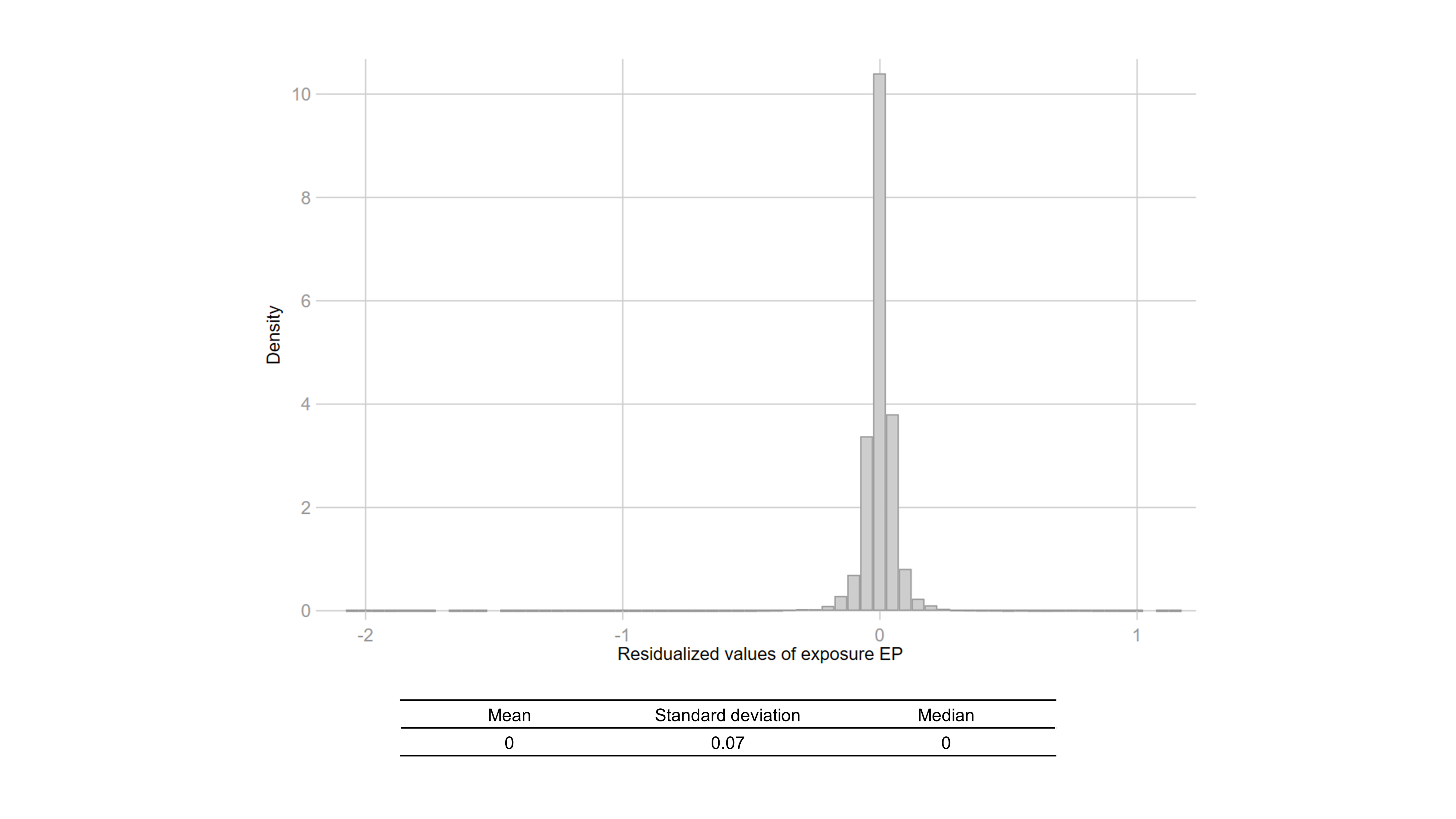

Supplement: Supplementary file 4 — Figure S3 [file HESR-55-823-s004.png]

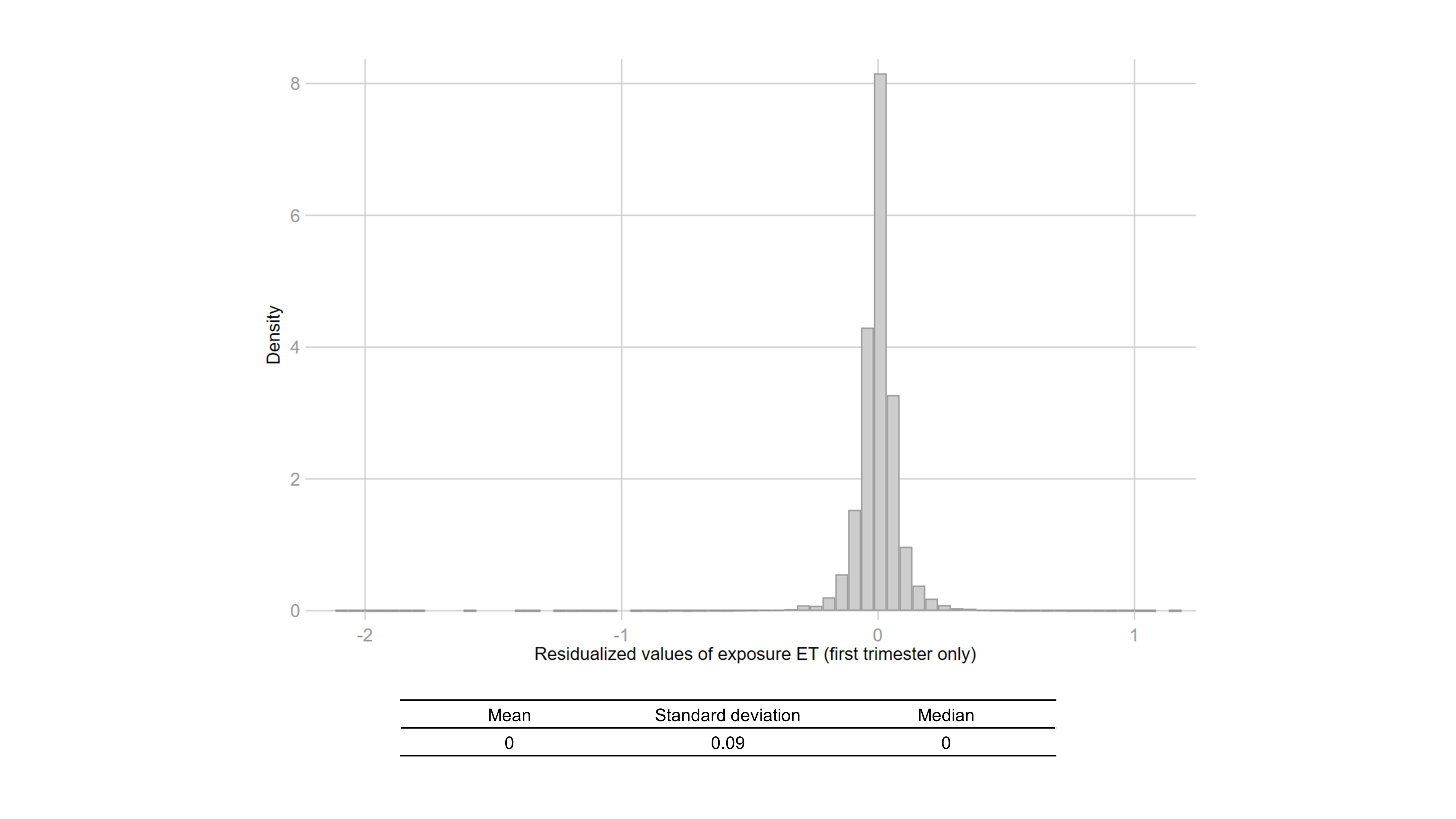

Supplement: Supplementary file 5 — Figure S4 [file HESR-55-823-s005.png]

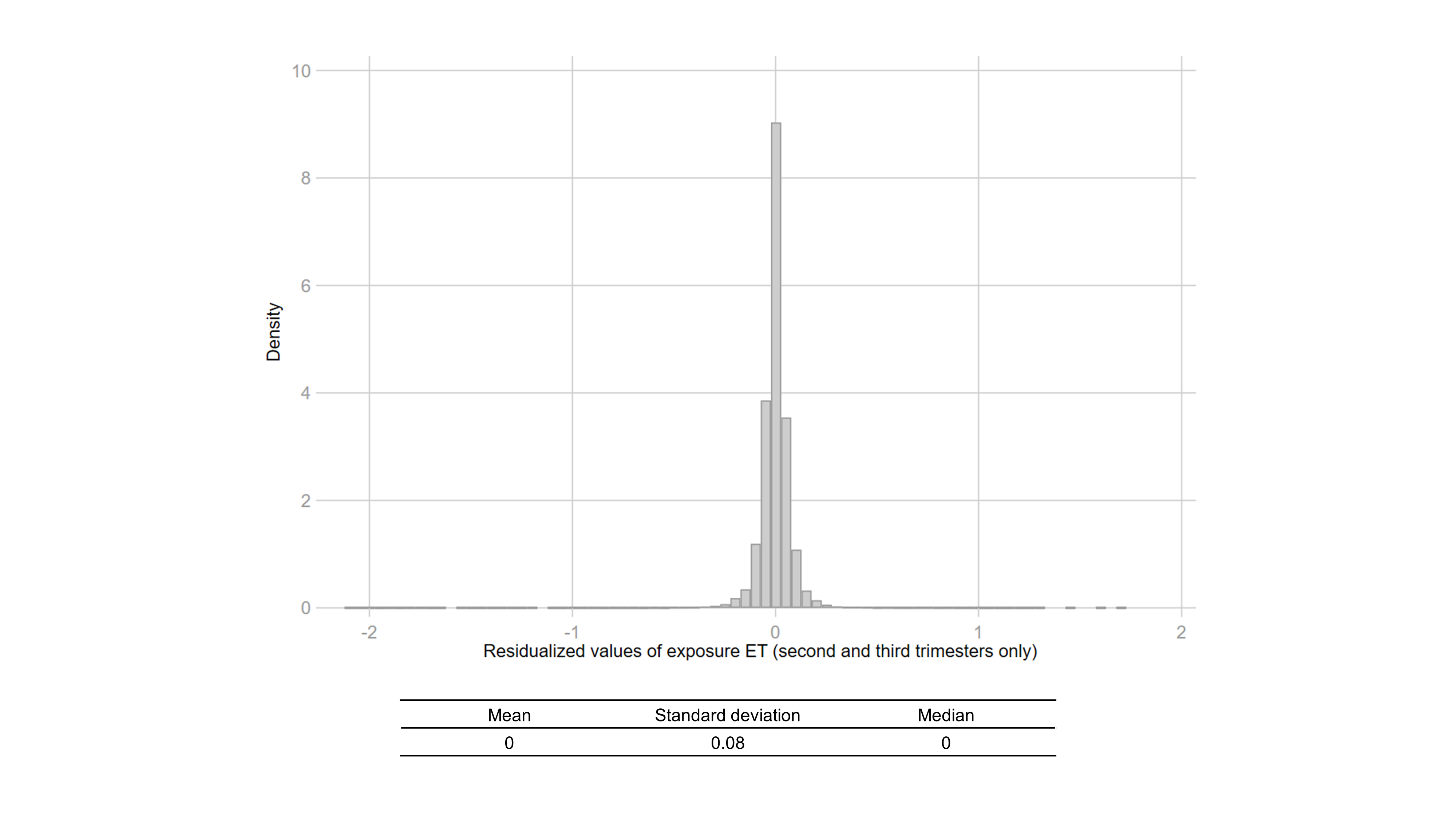

Supplement: Supplementary file 6 — Figure S5 [file HESR-55-823-s006.png]
