# Supplementary material for: In utero exposure to threat of evictions and preterm birth: Evidence from the United States
Source: Health Serv Res. 2020 Sep 25;55(Suppl 2):823–32. doi: 10.1111/1475-6773.13551 (PMC7518827; doi:10.1111/1475-6773.13551)
Supplement: Supplementary file 7 — Appendix S1 [file HESR-55-823-s007.doc]

# **Appendix I**

**Appendix Figure 1 States included in the analysis and the year from which they adopted the 2003 birth certificate revision**


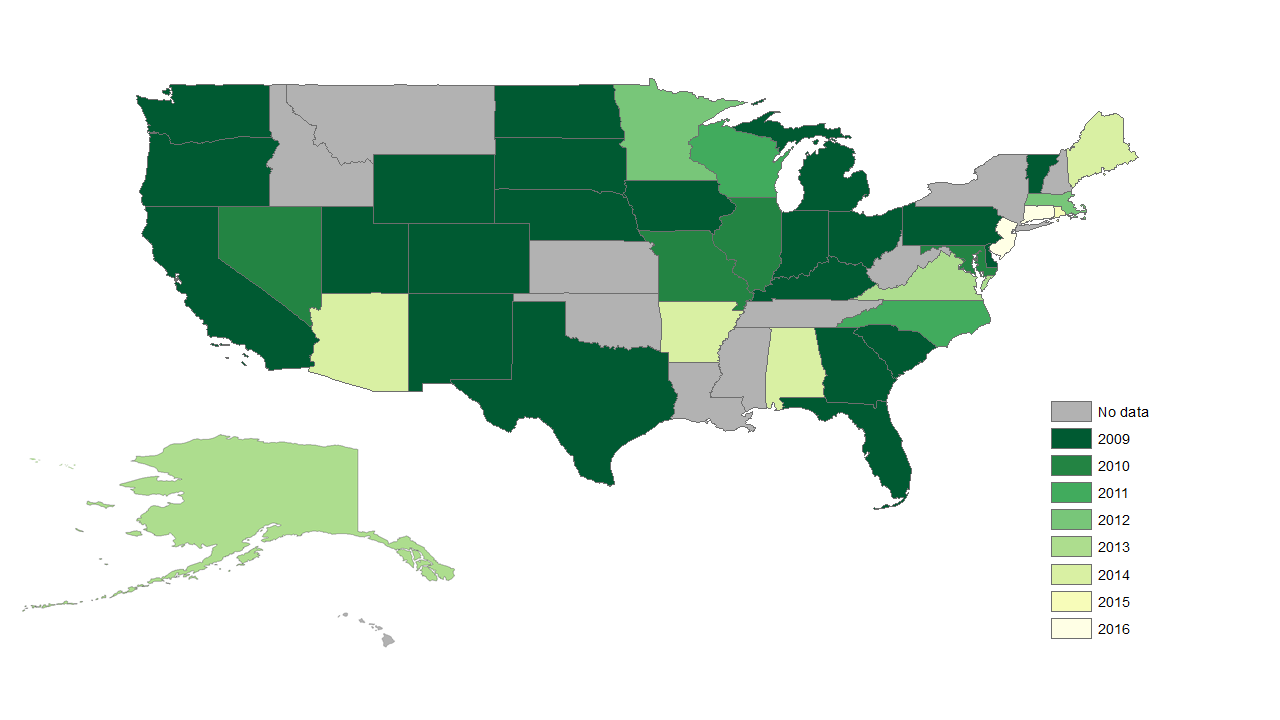
Note: For states that adopted the 2003 birth certificate revision mid-year, we used birth records starting from the subsequent year. For example, Rhode Island adopted the 2003 birth certificate revision some time after January 1, 2014. Therefore, we used birth records from Rhode Island starting 2015 only.

**Appendix II**

**Appendix Figure 2 Counties represented in our analysis and the length of the time series associated with each county**


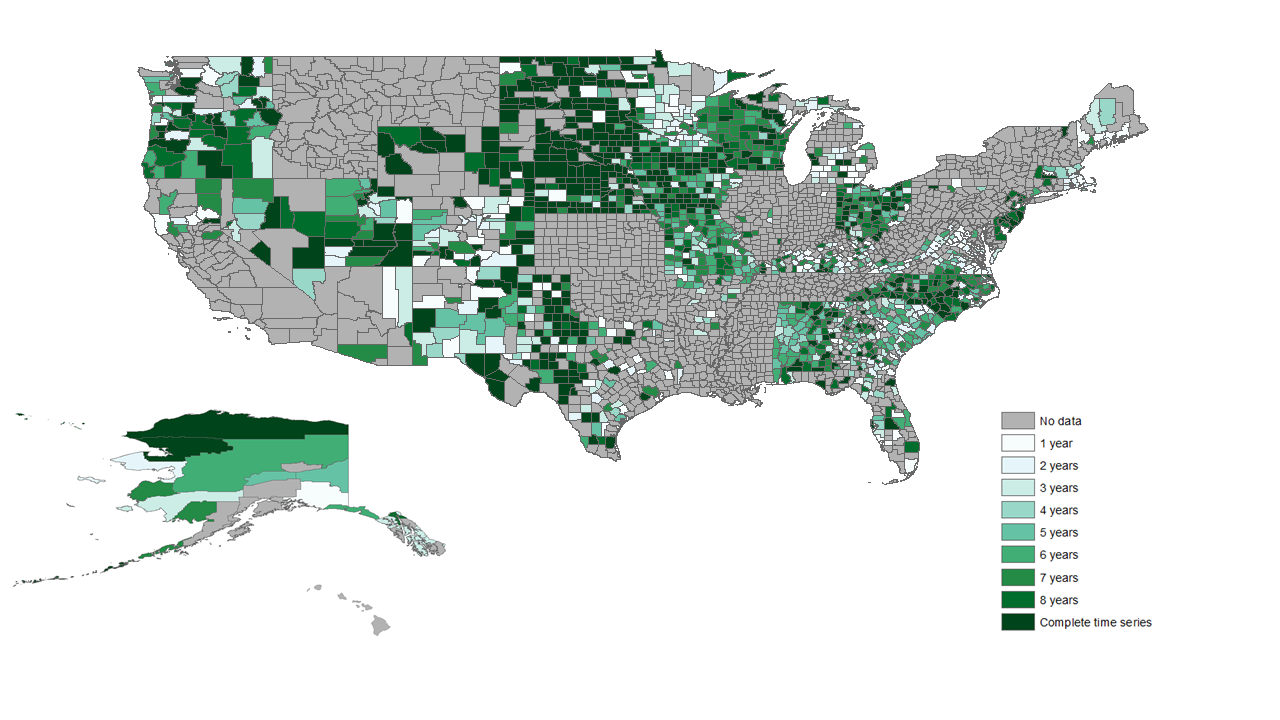


Notes: All counties colored in green are counties that have at least one year’s worth of eviction case filing data. The darker the shade of green, the longer the length of the time series for a given county. The legend displays the total number of years for which we have eviction case filing data for any given county.

# **Appendix III**

**Appendix Table 1 Frequency of missing data among variables in the analytic dataset**

|  | **Number of missing observations** | **Total number of observations** | **Percentage missing** |
| --- | --- | --- | --- |
| Birth weight in grams^1^ | 5,994 | 7,324,812 | 0.08 |
| Average eviction case filing during second and third trimesters | 2,636 | 7,324,812 | 0.04 |
| Average eviction case filing in the 9-month pre-pregnancy period | 42,257 | 7,324,812 | 0.58 |
| Parity | 44,289 | 7,324,812 | 0.6 |
| Method of payment | 111,537 | 7,324,812 | 1.52 |
| Average unemployment rate in county of residence during the entire pregnancy^*^ | 2,636 | 7,324,812 | 0.04 |
| Obstetric/clinical estimate of gestational length in weeks^2^ | 0 | 7,324,812 | 0 |
| Average eviction case filing during the entire pregnancy | 0 | 7,324,812 | 0 |
| Average eviction case filing during first trimester | 0 | 7,324,812 | 0 |
| Maternal age | 0 | 7,324,812 | 0 |
| Maternal education | 0 | 7,324,812 | 0 |
| Mother's race | 0 | 7,324,812 | 0 |
| Year of conception^3^ | 0 | 7,324,812 | 0 |
| Month of conception^3^ | 0 | 7,324,812 | 0 |
| County of residence^3^ | 0 | 7,324,812 | 0 |
| State of residence | 0 | 7,324,812 | 0 |
| Child's sex | 0 | 7,324,812 | 0 |
| Delivery type (singleton only) | 0 | 7,324,812 | 0 |
| Average poverty rate in county of residence in the year of conception^*^ | 0 | 7,324,812 | 0 |
| County of residence's urban/rural classification based on NCHS^*^ | 0 | 7,324,812 | 0 |

Notes: ^1^The low birth weight indicator variable was defined based on the birth weight variable. ^2^The preterm birth indicator variable was defined based on the obstetric/clinical estimate of gestational length. ^3^These variables were used to define the county-specific linear time trend. ^*^These variables were defined at the county-of-residence level.

# **Appendix IV**

**Appendix Table 2 Distribution of individual and county-level characteristics in 2009 and 2015 by tertiles defined using average exposure to eviction case filings over the duration of the pregnancy (exposure EP)**

|  | **2009** | | | **2015** | | |
| --- | --- | --- | --- | --- | --- | --- |
|  | **Low exposure tertile** | **Medium exposure tertile** | **High exposure tertile** | **Low exposure tertile** | **Medium exposure tertile** | **High exposure tertile** |
| **Individual-level** |  |  |  |  |  |  |
| *Mean age (years)* | 27.24 | 27.20 | 26.97 | 28.26 | 28.26 | 28.29 |
|  | (27.21 - 27.27) | (27.18 - 27.23) | (26.95 - 27) | (28.24 - 28.28) | (28.25 - 28.28) | (28.27 - 28.32) |
| *% of women with no High School* | 18.18% | 20.43% | 23.32% | 12.59% | 16.00% | 16.17% |
|  | (18.01% - 18.35%) | (20.25% - 20.61%) | (23.14% - 23.51%) | (12.48% - 12.7%) | (15.88% - 16.11%) | (16.03% - 16.3%) |
| *% of women with High School but no tertiary degree* | 47.6% | 46.9% | 48.4% | 45.9% | 47.2% | 46.3% |
|  | (47.34% - 47.78%) | (46.68% - 47.13%) | (48.16% - 48.6%) | (45.69% - 46.01%) | (47.02% - 47.34%) | (46.12% - 46.49%) |
| *% of women with a tertiary degree* | 34.3% | 32.7% | 28.3% | 41.6% | 36.8% | 37.5% |
|  | (34.05% - 34.47%) | (32.46% - 32.88%) | (28.1% - 28.5%) | (41.4% - 41.72%) | (36.67% - 36.97%) | (37.35% - 37.71%) |
| *% White (non-Hispanic)* | 69.8% | 54.7% | 39.4% | 73.0% | 50.7% | 41.2% |
|  | (69.57% - 69.96%) | (54.47% - 54.91%) | (39.18% - 39.61%) | (72.84% - 73.13%) | (50.55% - 50.86%) | (40.99% - 41.35%) |
| *% Black (non-Hispanic)* | 5.0% | 14.6% | 23.9% | 7.3% | 18.6% | 31.5% |
|  | (4.94% - 5.13%) | (14.45% - 14.77%) | (23.75% - 24.13%) | (7.21% - 7.38%) | (18.45% - 18.69%) | (31.35% - 31.69%) |
| *% Hispanic* | 18.0% | 25.5% | 31.1% | 11.7% | 24.9% | 20.7% |
|  | (17.83% - 18.16%) | (25.33% - 25.71%) | (30.86% - 31.27%) | (11.6% - 11.82%) | (24.81% - 25.08%) | (20.57% - 20.87%) |
| *% Other races* | 7.2% | 5.2% | 5.6% | 8.0% | 5.8% | 6.6% |
|  | (7.1% - 7.32%) | (5.08% - 5.28%) | (5.5% - 5.7%) | (7.92% - 8.09%) | (5.71% - 5.86%) | (6.5% - 6.68%) |
| *% paying for delivery using Medicaid* | 38.7% | 42.4% | 43.6% | 37.8% | 43.4% | 44.8% |
|  | (38.53% - 38.95%) | (42.2% - 42.64%) | (43.36% - 43.8%) | (37.65% - 37.97%) | (43.27% - 43.58%) | (44.63% - 45%) |
| **County-level** |  |  |  |  |  |  |
| *Average unemployment rate* | 7.5% | 7.8% | 7.7% | 5.0% | 5.4% | 5.7% |
|  | (7.48% - 7.51%) | (7.83% - 7.85%) | (7.64% - 7.66%) | (5.01% - 5.02%) | (5.36% - 5.36%) | (5.69% - 5.69%) |
| *Average poverty rate* | 12.1% | 13.5% | 15.1% | 14.2% | 16.6% | 17.4% |
|  | (12.06% - 12.1%) | (13.53% - 13.57%) | (15.08% - 15.1%) | (14.18% - 14.21%) | (16.59% - 16.61%) | (17.41% - 17.43%) |
| *% Metropolitan counties* | 65.1% | 95.7% | 98.5% | 67.7% | 93.8% | 96.9% |
|  | (64.85% - 65.26%) | (95.66% - 95.84%) | (98.49% - 98.6%) | (67.53% - 67.84%) | (93.74% - 93.9%) | (96.84% - 96.97%) |

Note: 95% confidence intervals in parentheses

# **Appendix V**

**Appendix Figure 3 Distribution of residuals after conditioning average eviction case filings over the duration of a pregnancy (exposure EP) on county of residence fixed effects, state-of-residence-year-month fixed effects, and county-specific linear time trend**


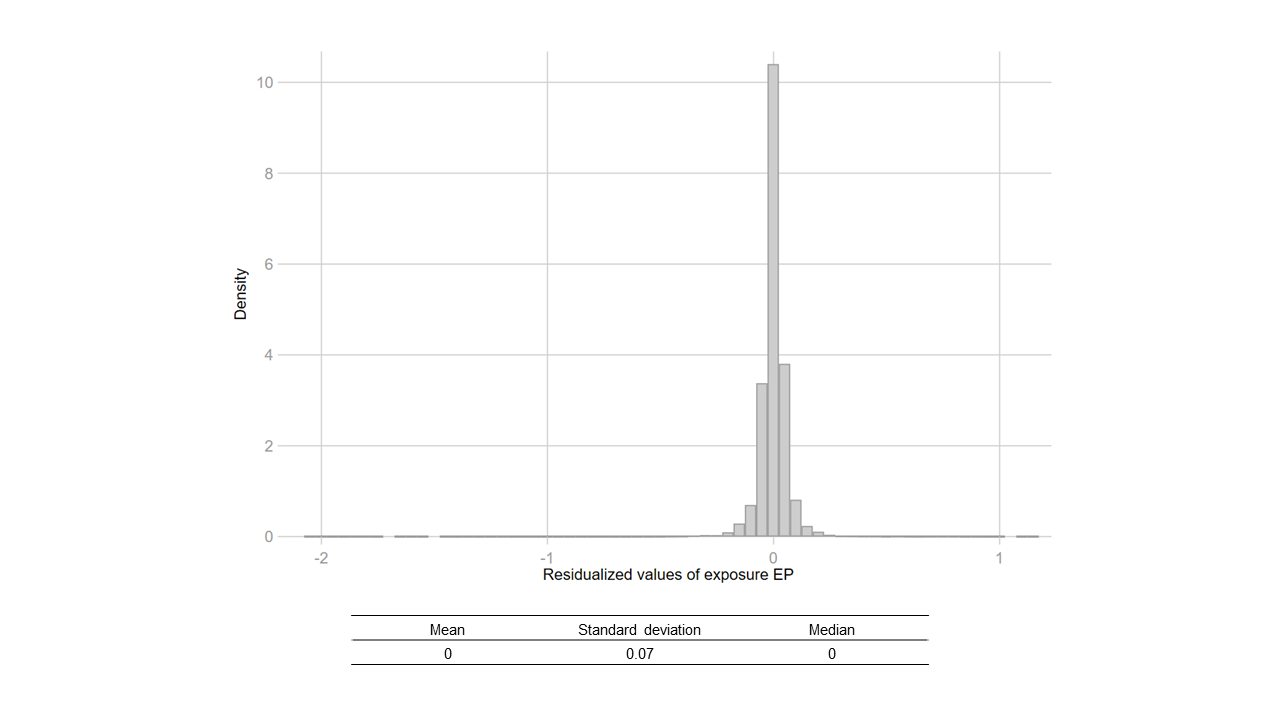


# **Appendix VI**

**Appendix Figure 4 Distribution of residuals after conditioning average eviction case filings in the first trimester (exposure ET) on county of residence fixed effects, state-of-residence-year-month fixed effects, and county-specific linear time trend**
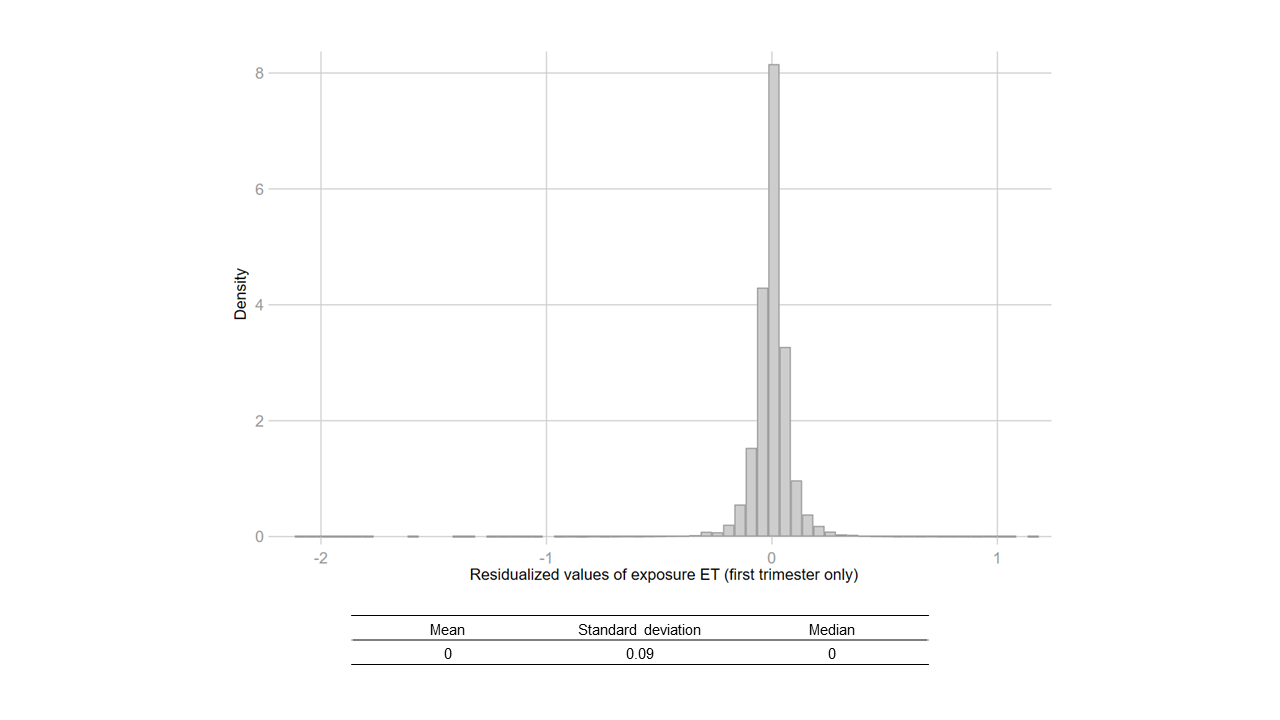


# **Appendix VII**

**Appendix Figure 5 Distribution of residuals after conditioning average eviction case filings in the second and third trimesters (exposure ET) on county of residence fixed effects, state-of-residence-year-month fixed effects, and county-specific linear time trend**


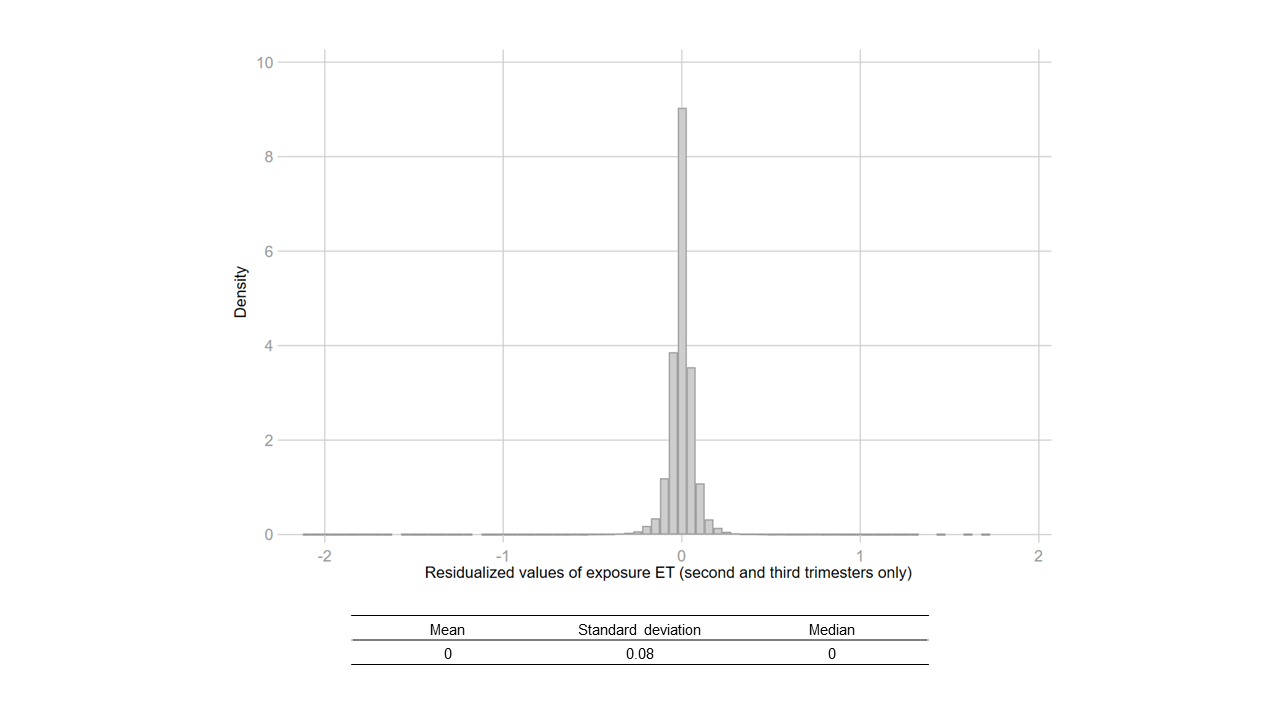


# **Appendix VIII**

**Appendix Table 3 Results from falsification check using exposure to average eviction case filings in the nine months prior to conception as a negative control**

|  | (1) | (2) | (3) | (4) |
| --- | --- | --- | --- | --- |
|  |  |  |  |  |
| Outcome | Preterm birth | Gestational length | Birth weight | Low birth weight |
| Z-score of average case filing in the nine months prior to conception | 0.12% points | 0.01 weeks | -1.15 grams | 0.13% points |
|  | (-0.55 - 0.8) | (-0.04 - 0.07) | (-9.57 - 7.26) | (-0.24 - 0.5) |
|  |  |  |  |  |
| Average value of the outcome in the analytic sample | 8.18% | 38.59 weeks | 3,298.56 grams | 6.57% |
|  |  |  |  |  |
|  |  |  |  |  |
| Average value of the outcome in the United States over the study period | 9.81% | 38.49 weeks | 3,269.06 grams | 8.07% |
|  |  |  |  |  |
|  |  |  |  |  |
| Observations | 7,324,812 | 7,324,812 | 7,324,812 | 7,324,812 |
|  |  |  |  |  |

Notes: 95% confidence intervals in parentheses constructed using clustered standard errors at the county-level. All results presented in the table are pooled estimates from Ordinary Least Squares regressions estimated on five imputed datasets. All models controlled for county of residence fixed effects, state-of-residence-year-and-month fixed effects, and a linear county-specific time trend. All models also controlled for threatened eviction exposure over the duration of the pregnancy, mother’s age, a quadratic age term, mother’s race, mother’s highest level of education, parity, child’s sex, method of payment for delivery, urban-rural classification of county of residence, county of residence’s annual poverty rate, and county of residence’s unstandardized, monthly unemployment rate.

# **Appendix IX**

**Appendix Table 4 Associations between exposure to average eviction case filings over the duration of the pregnancy (exposure EP) and birth outcomes in the verified cases sub-sample**

|  | (1) | (2) | (3) | (4) |
| --- | --- | --- | --- | --- |
| Outcome | Preterm birth | Gestational length | Birth weight | Low birth weight |
| Z-score of average case filing over pregnancy | 1.28% points | -0.06 weeks | -13.99 grams | 0.87% points |
|  | (0.07 - 2.49) | (-0.16 - 0.03) | (-33.45 - 5.47) | (0.04 - 1.7) |
|  |  |  |  |  |
| Average value of the outcome in the verified cases sample | 8.20% | 38.59 weeks | 3,297.33 grams | 6.58% |
|  |  |  |  |  |
|  |  |  |  |  |
| Average value of the outcome in the primary analytic sample | 8.18% | 38.59 weeks | 3,298.56 grams | 6.57% |
|  |  |  |  |  |
|  |  |  |  |  |
| Average value of the outcome in the United States over the study period | 9.81% | 38.49 weeks | 3,269.06 grams | 8.07% |
|  |  |  |  |  |
|  |  |  |  |  |
| Observations | 7,027,351 | 7,027,351 | 7,027,351 | 7,027,351 |
|  |  |  |  |  |

Notes: 95% confidence intervals in parentheses constructed using clustered standard errors at the county-level. All results presented in the table are pooled estimates from Ordinary Least Squares regressions estimated on five imputed datasets. All models controlled for county of residence fixed effects, state-of-residence-year-and-month fixed effects, and a linear county-specific time trend. All models also controlled for mother’s age, a quadratic age term, mother’s race, mother’s highest level of education, parity, child’s sex, method of payment for delivery, urban-rural classification of county of residence, county of residence’s annual poverty rate, and county of residence’s unstandardized, monthly unemployment rate.

# **Appendix X**

**Appendix Table 5 Associations between exposure to average eviction case filings over the duration of the pregnancy (exposure EP) and birth outcomes in the complete time series sub-sample**

|  | (1) | (2) | (3) | (4) |
| --- | --- | --- | --- | --- |
|  |  |  |  |  |
| Outcome | Preterm birth | Gestational length | Birth weight | Low birth weight |
| Z-score of average case filing over pregnancy | 0.45% points | -0.01 weeks | -4.74 grams | 0.31% points |
|  | (-0.25 - 1.14) | (-0.06 - 0.03) | (-17.51 - 8.03) | (-0.24 - 0.86) |
|  |  |  |  |  |
| Average value of the outcome among counties with complete panel of exposure data | 7.89% | 38.63 weeks | 3,310.39 grams | 6.34% |
|  |  |  |  |  |
|  |  |  |  |  |
| Average value of the outcome in the primary analytic sample | 8.18% | 38.59 weeks | 3,298.56 grams | 6.57% |
|  |  |  |  |  |
|  |  |  |  |  |
| Average value of the outcome in the United States over the study period | 9.81% | 38.49 weeks | 3,269.06 grams | 8.07% |
|  |  |  |  |  |
|  |  |  |  |  |
| Observations | 3,254,301 | 3,254,301 | 3,254,301 | 3,254,301 |
|  |  |  |  |  |

Notes: 95% confidence intervals in parentheses constructed using clustered standard errors at the county-level. All results presented in the table are pooled estimates from Ordinary Least Squares regressions estimated on five imputed datasets. All models controlled for county of residence fixed effects, state-of-residence-year-and-month fixed effects, and a linear county-specific time trend. All models also controlled for mother’s age, a quadratic age term, mother’s race, mother’s highest level of education, parity, child’s sex, method of payment for delivery, urban-rural classification of county of residence, county of residence’s annual poverty rate, and county of residence’s unstandardized, monthly unemployment rate.

# **Appendix XI**

**Appendix Table 6 Associations between exposure to average eviction case filings over the duration of the pregnancy (exposure EP) and birth outcomes in the five-year time series sub-sample**

|  | (1) | (2) | (3) | (4) |
| --- | --- | --- | --- | --- |
|  |  |  |  |  |
| Outcome | Preterm birth | Gestational length | Birth weight | Low birth weight |
| Z-score of average case filing over pregnancy | 1.42% points | -0.08 weeks | -16.88 grams | 0.92% points |
|  | (0.5 - 2.34) | (-0.14 - -0.01) | (-32.09 - -1.67) | (0.3 - 1.55) |
|  |  |  |  |  |
| Average value of the outcome among counties with exposure information for five years or more | 8.11% | 38.60 weeks | 3,301.84 grams | 6.49% |
|  |  |  |  |  |
|  |  |  |  |  |
| Average value of the outcome in the primary analytic sample | 8.18% | 38.59 weeks | 3,298.56 grams | 6.57% |
|  |  |  |  |  |
|  |  |  |  |  |
| Average value of the outcome in the United States over the study period | 9.81% | 38.49 weeks | 3,269.06 grams | 8.07% |
|  |  |  |  |  |
|  |  |  |  |  |
| Observations | 6,364,818 | 6,364,818 | 6,364,818 | 6,364,818 |
|  |  |  |  |  |

Notes: 95% confidence intervals in parentheses constructed using clustered standard errors at the county-level. All results presented in the table are pooled estimates from Ordinary Least Squares regressions estimated on five imputed datasets. All models controlled for county of residence fixed effects, state-of-residence-year-and-month fixed effects, and a linear county-specific time trend. All models also controlled for mother’s age, a quadratic age term, mother’s race, mother’s highest level of education, parity, child’s sex, method of payment for delivery, urban-rural classification of county of residence, county of residence’s annual poverty rate, and county of residence’s unstandardized, monthly unemployment rate.

# **Appendix XII**

**Appendix Table 7 Associations between exposure to average eviction case filings** by pregnancy trimester (exposure ET) and birth outcomes in the verified cases sub-sample

|  | (1) | (2) | (3) | (4) |
| --- | --- | --- | --- | --- |
|  |  |  |  |  |
| Outcome | Preterm birth | Gestational length | Birth weight | Low birth weight |
| Z-score of average case filing in the first trimester | -0.03% points | 0.000 weeks | 0.92 grams | 0.040% points |
|  | (-0.47 - 0.41) | (-0.03 - 0.03) | (-5.88 - 7.72) | (-0.31 - 0.39) |
| Z-score of average case filing in the second and third trimesters | 0.96% points | -0.04 weeks | -10.02 grams | 0.648% points |
|  | (-0.02 - 1.93) | (-0.13 - 0.05) | (-26.08 - 6.05) | (-0.07 - 1.36) |
|  |  |  |  |  |
| Average value of the outcome in the verified cases sample | 8.20% | 38.59 weeks | 3,297.33 grams | 6.58% |
|  |  |  |  |  |
|  |  |  |  |  |
| Average value of the outcome in the primary analytic sample | 8.18% | 38.59 weeks | 3,298.56 grams | 6.57% |
|  |  |  |  |  |
|  |  |  |  |  |
| Average value of the outcome in the United States over the study period | 9.81% | 38.49 weeks | 3,269.06 grams | 8.07% |
|  |  |  |  |  |
|  |  |  |  |  |
| Observations | 7,027,351 | 7,027,351 | 7,027,351 | 7,027,351 |
|  |  |  |  |  |

Notes: 95% confidence intervals in parentheses constructed using clustered standard errors at the county-level. All results presented in the table are pooled estimates from Ordinary Least Squares regressions estimated on five imputed datasets. All models controlled for county of residence fixed effects, state-of-residence-year-and-month fixed effects, and a linear county-specific time trend. All models also controlled for mother’s age, a quadratic age term, mother’s race, mother’s highest level of education, parity, child’s sex, method of payment for delivery, urban-rural classification of county of residence, county of residence’s annual poverty rate, and county of residence’s unstandardized, monthly unemployment rate.

# **Appendix XIII**

**Appendix Table 8 Associations between exposure to average eviction case filings by pregnancy trimester (exposure ET) and birth outcomes in the complete time series** sub-sample

|  | (1) | (2) | (3) | (4) |
| --- | --- | --- | --- | --- |
|  |  |  |  |  |
| Outcome | Preterm birth | Gestational length | Birth weight | Low birth weight |
| Z-score of average case filing in the first trimester | -0.02% points | -0.009 weeks | -3.10 grams | 0.07% points |
|  | (-0.33 - 0.3) | (-0.04 - 0.02) | (-10.08 - 3.87) | (-0.19 - 0.32) |
| Z-score of average case filing in the second and third trimesters | 0.25% points | 0.01 weeks | 0.84 grams | 0.15% points |
|  | (-0.28 - 0.77) | (-0.04 - 0.05) | (-8.94 - 10.63) | (-0.31 - 0.61) |
|  |  |  |  |  |
| Average value of the outcome among counties with complete panel of exposure data | 7.89% | 38.63 weeks | 3,310.39 grams | 6.34% |
|  |  |  |  |  |
|  |  |  |  |  |
| Average value of the outcome in the primary analytic sample | 8.18% | 38.59 weeks | 3,298.56 grams | 6.57% |
|  |  |  |  |  |
|  |  |  |  |  |
| Average value of the outcome in the United States over the study period | 9.81% | 38.49 weeks | 3,269.06 grams | 8.07% |
|  |  |  |  |  |
|  |  |  |  |  |
| Observations | 3,254,301 | 3,254,301 | 3,254,301 | 3,254,301 |
|  |  |  |  |  |

Notes: 95% confidence intervals in parentheses constructed using clustered standard errors at the county-level. All results presented in the table are pooled estimates from Ordinary Least Squares regressions estimated on five imputed datasets. All models controlled for county of residence fixed effects, state-of-residence-year-and-month fixed effects, and a linear county-specific time trend. All models also controlled for mother’s age, a quadratic age term, mother’s race, mother’s highest level of education, parity, child’s sex, method of payment for delivery, urban-rural classification of county of residence, county of residence’s annual poverty rate, and county of residence’s unstandardized, monthly unemployment rate.

# **Appendix XIV**

**Appendix Table 9 Associations between exposure to average eviction case filings by pregnancy trimester (exposure ET) and birth outcomes in the five-year time series sub-sample**

|  | (1) | (2) | (3) | (4) |
| --- | --- | --- | --- | --- |
|  |  |  |  |  |
| Outcome | Preterm birth | Gestational length | Birth weight | Low birth weight |
| Z-score of average case filing in the first trimester | 0.08% points | -0.005 weeks | -0.24 grams | 0.06% points |
|  | (-0.32 - 0.48) | (-0.04 - 0.03) | (-7.24 - 6.77) | (-0.26 - 0.38) |
| Z-score of average case filing in the second and third trimesters | 0.88% points | -0.04 weeks | -10.20 grams | 0.59% points |
|  | (0.15 - 1.62) | (-0.1 - 0.02) | (-22.07 - 1.66) | (0.02 - 1.15) |
|  |  |  |  |  |
| Average value of the outcome among counties with exposure information for five years or more | 8.11% | 38.60 weeks | 3,301.84 grams | 6.49% |
|  |  |  |  |  |
|  |  |  |  |  |
| Average value of the outcome in the primary analytic sample | 8.18% | 38.59 weeks | 3,298.56 grams | 6.57% |
|  |  |  |  |  |
|  |  |  |  |  |
| Average value of the outcome in the United States over the study period | 9.81% | 38.49 weeks | 3,269.06 grams | 8.07% |
|  |  |  |  |  |
|  |  |  |  |  |
| Observations | 6,364,818 | 6,364,818 | 6,364,818 | 6,364,818 |
|  |  |  |  |  |

Notes: 95% confidence intervals in parentheses constructed using clustered standard errors at the county-level. All results presented in the table are pooled estimates from Ordinary Least Squares regressions estimated on five imputed datasets. All models controlled for county of residence fixed effects, state-of-residence-year-and-month fixed effects, and a linear county-specific time trend. All models also controlled for mother’s age, a quadratic age term, mother’s race, mother’s highest level of education, parity, child’s sex, method of payment for delivery, urban-rural classification of county of residence, county of residence’s annual poverty rate, and county of residence’s unstandardized, monthly unemployment rate.

# **Appendix XV**

**Appendix Table 10 Associations between exposure to average eviction case filings** over the duration of a pregnancy (exposure EP) and preterm birth by racial sub-groups

|  | (1) | (2) | (3) | (4) |
| --- | --- | --- | --- | --- |
|  |  |  |  |  |
| Racial category | White non-Hispanic | Black non-Hispanic | Hispanic | Other races |
| Z-score of average case filing over pregnancy | 1.06% points | 2.13% points | 0.69% points | 1.27% points |
|  | (0.17 - 1.95) | (-0.06 - 4.33) | (-1.05 - 2.43) | (-0.64 - 3.18) |
|  |  |  |  |  |
| Average proportion or preterm birth in the analytic sample | 7.18% | 11.59% | 8.14% | 7.84% |
|  |  |  |  |  |
|  |  |  |  |  |
| Observations in analytic sample | 4,100,995 | 1,277,406 | 1,463,664 | 482,747 |
|  |  |  |  |  |

Notes: All coefficient estimates reflect percentage point change in the risk of preterm birth for a standard deviation change in average case filings over a pregnancy. 95% confidence intervals in parentheses constructed using clustered standard errors at the county-level. All results presented in the table are pooled estimates from Ordinary Least Squares regressions estimated on five imputed datasets. All models controlled for county of residence fixed effects, state-of-residence-year-and-month fixed effects, and a linear county-specific time trend. All models also controlled for mother’s age, a quadratic age term, mother’s highest level of education, parity, child’s sex, method of payment for delivery, urban-rural classification of county of residence, county of residence’s annual poverty rate, and county of residence’s unstandardized, monthly unemployment rate.

# **Appendix XVI**

**Appendix Table 11 Associations between exposure to average eviction case filings over the duration of a pregnancy (exposure EP) and gestational length** by racial sub-groups

|  | (1) | (2) | (3) | (4) |
| --- | --- | --- | --- | --- |
|  |  |  |  |  |
| Racial category | White non-Hispanic | Black non-Hispanic | Hispanic | Other races |
| Z-score of average case filing over pregnancy | -0.07 weeks | -0.16 weeks | 0.01 weeks | 0.02 weeks |
|  | (-0.14 - -0.01) | (-0.35 - 0.03) | (-0.13 - 0.14) | (-0.17 - 0.21) |
|  |  |  |  |  |
| Average length of gestation in the analytic sample | 38.71 weeks | 38.25 weeks | 38.58 weeks | 38.57 weeks |
|  |  |  |  |  |
|  |  |  |  |  |
| Observations in analytic sample | 4,100,995 | 1,277,406 | 1,463,664 | 482,747 |
|  |  |  |  |  |

Notes: All coefficient estimates reflect change in gestational length measured in weeks for a standard deviation change in average case filings over a pregnancy. 95% confidence intervals in parentheses constructed using clustered standard errors at the county-level. All results presented in the table are pooled estimates from Ordinary Least Squares regressions estimated on five imputed datasets. All models controlled for county of residence fixed effects, state-of-residence-year-and-month fixed effects, and a linear county-specific time trend. All models also controlled for mother’s age, a quadratic age term, mother’s highest level of education, parity, child’s sex, method of payment for delivery, urban-rural classification of county of residence, county of residence’s annual poverty rate, and county of residence’s unstandardized, monthly unemployment rate.

# **Appendix XVII**

**Appendix Table 12 Associations between exposure to average eviction case filings over the duration of a pregnancy (exposure EP) and birth weight by racial sub-groups**

|  | (1) | (2) | (3) | (4) |
| --- | --- | --- | --- | --- |
|  |  |  |  |  |
| Racial category | White non-Hispanic | Black non-Hispanic | Hispanic | Other races |
| Z-score of average case filing over pregnancy | -14.33 grams | -27.38 grams | -11.27 grams | 10.38 grams |
|  | (-28.16 - -0.51) | (-60.3 - 5.55) | (-39.95 - 17.41) | (-29.67 - 50.43) |
|  |  |  |  |  |
| Average birth weight in the analytic sample (grams) | 3,369.33 grams | 3,111.65 grams | 3,288.01 grams | 3,223.35 grams |
|  |  |  |  |  |
|  |  |  |  |  |
| Observations in analytic sample | 4,100,995 | 1,277,406 | 1,463,664 | 482,747 |
|  |  |  |  |  |

Notes: All coefficient estimates reflect change in birth weight measured in grams for a standard deviation change in average case filings over a pregnancy. 95% confidence intervals in parentheses constructed using clustered standard errors at the county-level. All results presented in the table are pooled estimates from Ordinary Least Squares regressions estimated on five imputed datasets. All models controlled for county of residence fixed effects, state-of-residence-year-and-month fixed effects, and a linear county-specific time trend. All models also controlled for mother’s age, a quadratic age term, mother’s highest level of education, parity, child’s sex, method of payment for delivery, urban-rural classification of county of residence, county of residence’s annual poverty rate, and county of residence’s unstandardized, monthly unemployment rate.

# **Appendix XVIII**

**Appendix Table 13 Associations between exposure to average eviction case filings over the duration of a pregnancy (exposure EP) and low birth weight by racial sub-groups**

|  | (1) | (2) | (3) | (4) |
| --- | --- | --- | --- | --- |
|  |  |  |  |  |
| Racial category | White non-Hispanic | Black non-Hispanic | Hispanic | Other races |
| Z-score of average case filing over pregnancy | 0.88% points | 1.69% points | 0.12% points | -0.57% points |
|  | (0.25 - 1.5) | (0.16 - 3.22) | (-0.88 - 1.11) | (-2.1 - 0.96) |
|  |  |  |  |  |
| Average proportion of low birth weight in the analytic sample | 5.19% | 11.34% | 6.11% | 7.09% |
|  |  |  |  |  |
|  |  |  |  |  |
| Observations in analytic sample | 4,100,995 | 1,277,406 | 1,463,664 | 482,747 |
|  |  |  |  |  |

Notes: All coefficient estimates reflect percentage point change in the risk of low birth weight for a standard deviation change in average case filings over a pregnancy. 95% confidence intervals in parentheses constructed using clustered standard errors at the county-level. All results presented in the table are pooled estimates from Ordinary Least Squares regressions estimated on five imputed datasets. All models controlled for county of residence fixed effects, state-of-residence-year-and-month fixed effects, and a linear county-specific time trend. All models also controlled for mother’s age, a quadratic age term, mother’s highest level of education, parity, child’s sex, method of payment for delivery, urban-rural classification of county of residence, county of residence’s annual poverty rate, and county of residence’s unstandardized, monthly unemployment rate.

# **Appendix XIX**

**Appendix Table 14 Associations between exposure to average eviction case filings over the duration of the pregnancy (exposure EP) and birth outcomes among women who paid for their delivery using Medicaid**

|  | (1) | (2) | (3) | (4) |
| --- | --- | --- | --- | --- |
|  |  |  |  |  |
| Outcome | Preterm birth | Gestational length | Birth weight | Low birth weight |
| Z-score of average case filing over pregnancy | 1.03% points | -0.03 weeks | -8.43 grams | 0.63% points |
|  | (-0.12 - 2.18) | (-0.13 - 0.06) | (-28.75 - 11.89) | (-0.19 - 1.45) |
|  |  |  |  |  |
| Average value of the outcome among women using Medicaid to pay for deliveries | 9.37% | 38.48 weeks | 3,225.87 grams | 8.20% |
|  |  |  |  |  |
|  |  |  |  |  |
| Observations in analytic sample | 3,083,408 | 3,083,408 | 3,083,408 | 3,083,408 |
|  |  |  |  |  |

Notes: 95% confidence intervals in parentheses constructed using clustered standard errors at the county-level. All results presented in the table are pooled estimates from Ordinary Least Squares regressions estimated on five imputed datasets. All models controlled for county of residence fixed effects, state-of-residence-year-and-month fixed effects, and a linear county-specific time trend. All models also controlled for mother’s age, a quadratic age term, mother’s race, mother’s highest level of education, parity, child’s sex, urban-rural classification of county of residence, county of residence’s annual poverty rate, and county of residence’s unstandardized, monthly unemployment rate.

# **Appendix XX**

**Appendix Table 15 Associations between exposure to average eviction case filings by pregnancy trimester (exposure ET) and preterm birth by racial sub-groups**

|  | (1) | (2) | (3) | (4) |
| --- | --- | --- | --- | --- |
|  |  |  |  |  |
| Racial category | White non-Hispanic | Black non-Hispanic | Hispanic | Other races |
| Z-score of average case filing in the first trimester | 0.18% points | 0.37% points | -0.61% points | -0.67% points |
|  | (-0.23 - 0.6) | (-0.51 - 1.25) | (-1.65 - 0.43) | (-1.76 - 0.42) |
| Z-score of average case filing in the second and third trimesters | 0.62% points | 1.86% points | 1.10% points | 1.96% points |
|  | (-0.07 - 1.31) | (-0.02 - 3.73) | (-0.05 - 2.26) | (0.16 - 3.75) |
|  |  |  |  |  |
| Average proportion or preterm birth in the analytic sample | 7.18% | 11.59% | 8.14% | 7.84% |
|  |  |  |  |  |
|  |  |  |  |  |
| Observations in analytic sample | 4,100,995 | 1,277,406 | 1,463,664 | 482,747 |
|  |  |  |  |  |

Notes: All coefficient estimates reflect percentage point change in the risk of preterm birth for a standard deviation change in average case filings over a pregnancy. 95% confidence intervals in parentheses constructed using clustered standard errors at the county-level. All results presented in the table are pooled estimates from Ordinary Least Squares regressions estimated on five imputed datasets. All models controlled for county of residence fixed effects, state-of-residence-year-and-month fixed effects, and a linear county-specific time trend. All models also controlled for mother’s age, a quadratic age term, mother’s highest level of education, parity, child’s sex, method of payment for delivery, urban-rural classification of county of residence, county of residence’s annual poverty rate, and county of residence’s unstandardized, monthly unemployment rate.

# **Appendix XXI**

**Appendix Table 16 Associations between exposure to average eviction case filings by pregnancy trimester (exposure ET) and gestational length by racial sub-groups**

|  | (1) | (2) | (3) | (4) |
| --- | --- | --- | --- | --- |
|  |  |  |  |  |
| Racial category | White non-Hispanic | Black non-Hispanic | Hispanic | Other races |
| Z-score of average case filing in the first trimester | -0.01 weeks | -0.04 weeks | 0.03 weeks | 0.04 weeks |
|  | (-0.04 - 0.02) | (-0.11 - 0.04) | (-0.04 - 0.09) | (-0.04 - 0.12) |
| Z-score of average case filing in the second and third trimesters | -0.04 weeks | -0.13 weeks | 0.00 weeks | -0.05 weeks |
|  | (-0.1 - 0.01) | (-0.32 - 0.06) | (-0.11 - 0.11) | (-0.21 - 0.12) |
|  |  |  |  |  |
| Average length of gestation in the analytic sample | 38.71 weeks | 38.25 weeks | 38.58 weeks | 38.57 weeks |
|  |  |  |  |  |
|  |  |  |  |  |
| Observations in analytic sample | 4,100,995 | 1,277,406 | 1,463,664 | 482,747 |
|  |  |  |  |  |

Notes: All coefficient estimates reflect change in gestational length measured in weeks for a standard deviation change in average case filings over a pregnancy. 95% confidence intervals in parentheses constructed using clustered standard errors at the county-level. All results presented in the table are pooled estimates from Ordinary Least Squares regressions estimated on five imputed datasets. All models controlled for county of residence fixed effects, state-of-residence-year-and-month fixed effects, and a linear county-specific time trend. All models also controlled for mother’s age, a quadratic age term, mother’s highest level of education, parity, child’s sex, method of payment for delivery, urban-rural classification of county of residence, county of residence’s annual poverty rate, and county of residence’s unstandardized, monthly unemployment rate.

# **Appendix XXII**

**Appendix Table 17 Associations between exposure to average eviction case filings by pregnancy trimester (exposure ET) and birth weight by racial sub-groups**

|  | (1) | (2) | (3) | (4) |
| --- | --- | --- | --- | --- |
|  |  |  |  |  |
| Racial category | White non-Hispanic | Black non-Hispanic | Hispanic | Other races |
| Z-score of average case filing in the first trimester | -1.43 grams | -5.45 grams | 3.07 grams | 11.51 grams |
|  | (-8.74 - 5.88) | (-19.44 - 8.53) | (-10.12 - 16.26) | (-9.02 - 32.05) |
| Z-score of average case filing in the second and third trimesters | -9.21 grams | -23.61 grams | -10.97 grams | -3.03 grams |
|  | (-20.95 - 2.53) | (-56.64 - 9.42) | (-32.37 - 10.43) | (-37.66 - 31.6) |
|  |  |  |  |  |
| Average birth weight in the analytic sample (grams) | 3,369.33 grams | 3,111.65 grams | 3,288.01 grams | 3,223.35 grams |
|  |  |  |  |  |
|  |  |  |  |  |
| Observations in analytic sample | 4,100,995 | 1,277,406 | 1,463,664 | 482,747 |
|  |  |  |  |  |

Notes: All coefficient estimates reflect change in birth weight measured in grams for a standard deviation change in average case filings over a pregnancy. 95% confidence intervals in parentheses constructed using clustered standard errors at the county-level. All results presented in the table are pooled estimates from Ordinary Least Squares regressions estimated on five imputed datasets. All models controlled for county of residence fixed effects, state-of-residence-year-and-month fixed effects, and a linear county-specific time trend. All models also controlled for mother’s age, a quadratic age term, mother’s highest level of education, parity, child’s sex, method of payment for delivery, urban-rural classification of county of residence, county of residence’s annual poverty rate, and county of residence’s unstandardized, monthly unemployment rate.

# **Appendix XXIII**

**Appendix Table 18 Associations between exposure to average eviction case filings by pregnancy trimester (exposure ET) and low birth weight by racial sub-groups**

|  | (1) | (2) | (3) | (4) |
| --- | --- | --- | --- | --- |
|  |  |  |  |  |
| Racial category | White non-Hispanic | Black non-Hispanic | Hispanic | Other races |
| Z-score of average case filing in the first trimester | 0.19% points | 0.35% points | -0.34% points | -0.25% points |
|  | (-0.09 - 0.46) | (-0.41 - 1.11) | (-0.96 - 0.28) | (-1.24 - 0.74) |
| Z-score of average case filing in the second and third trimesters | 0.56% points | 1.44% points | 0.38% points | -0.24% points |
|  | (0.03 - 1.09) | (-0.12 - 2.99) | (-0.44 - 1.2) | (-1.85 - 1.37) |
|  |  |  |  |  |
| Average proportion of low birth weight in the analytic sample | 5.19% | 11.34% | 6.11% | 7.09% |
|  |  |  |  |  |
|  |  |  |  |  |
| Observations in analytic sample | 4,100,995 | 1,277,406 | 1,463,664 | 482,747 |
|  |  |  |  |  |

Notes: All coefficient estimates reflect percentage point change in the risk of low birth weight for a standard deviation change in average case filings over a pregnancy. 95% confidence intervals in parentheses constructed using clustered standard errors at the county-level. All results presented in the table are pooled estimates from Ordinary Least Squares regressions estimated on five imputed datasets. All models controlled for county of residence fixed effects, state-of-residence-year-and-month fixed effects, and a linear county-specific time trend. All models also controlled for mother’s age, a quadratic age term, mother’s highest level of education, parity, child’s sex, method of payment for delivery, urban-rural classification of county of residence, county of residence’s annual poverty rate, and county of residence’s unstandardized, monthly unemployment rate.

# **Appendix XXIV**

**Appendix Table 19 Associations between exposure to average eviction case filings by pregnancy trimester (exposure ET) and birth outcomes among women who paid for their delivery using Medicaid**

|  | (1) | (2) | (3) | (4) |
| --- | --- | --- | --- | --- |
|  |  |  |  |  |
| Outcome | Preterm birth | Gestational length | Birth weight | Low birth weight |
| Z-score of average case filing in the first trimester | -0.05% points | 0.02 weeks | 7.05 grams | -0.26% points |
|  | (-0.61 - 0.5) | (-0.02 - 0.06) | (-2.27 - 16.38) | (-0.73 - 0.21) |
| Z-score of average case filing in the second and third trimesters | 1.06% points | -0.06 weeks | -15.84 grams | 0.94% points |
|  | (0.05 - 2.08) | (-0.15 - 0.04) | (-33.66 - 1.98) | (0.1 - 1.78) |
|  |  |  |  |  |
| Average value of the outcome among women using Medicaid to pay for deliveries | 9.37% | 38.48 weeks | 3,225.87 grams | 8.20% |
|  |  |  |  |  |
|  |  |  |  |  |
| Observations in analytic sample | 3,083,408 | 3,083,408 | 3,083,408 | 3,083,408 |
|  |  |  |  |  |

Notes: 95% confidence intervals in parentheses constructed using clustered standard errors at the county-level. All results presented in the table are pooled estimates from Ordinary Least Squares regressions estimated on five imputed datasets. All models controlled for county of residence fixed effects, state-of-residence-year-and-month fixed effects, and a linear county-specific time trend. All models also controlled for mother’s age, a quadratic age term, mother’s race, mother’s highest level of education, parity, child’s sex, urban-rural classification of county of residence, county of residence’s annual poverty rate, and county of residence’s unstandardized, monthly unemployment rate.
